# Application of Machine Learning Classification to Improve the Performance of Vancomycin Therapeutic Drug Monitoring

**DOI:** 10.3390/pharmaceutics14051023

**Published:** 2022-05-09

**Authors:** Sooyoung Lee, Moonsik Song, Jongdae Han, Donghwan Lee, Bo-Hyung Kim

**Affiliations:** 1 Department of Life and Nanopharmaceutical Sciences, Graduate School, Kyung Hee University, Seoul 02447, Korea; ls009@khu.ac.kr; 2 Department of Biomedical Science and Technology, Graduate School, Kyung Hee University, Seoul 02447, Korea; sikso1897@gmail.com; 3 Department of Computer Science, Sangmyung University, Seoul 03016, Korea; elvenwhite@smu.ac.kr; 4 Department of Statistics, Ewha Womans University, Seoul 03760, Korea; 5 Department of Clinical Pharmacology and Therapeutics, Kyung Hee University Medical Center, Seoul 02447, Korea; 6 Department of Biomedical and Pharmaceutical Sciences, Graduate School, Kyung Hee University, Seoul 02447, Korea; 7 East-West Medical Research Institute, Kyung Hee University, Seoul 02447, Korea

**Keywords:** population pharmacokinetics, simulation, Bayesian method, XGBoost, classifier

## Abstract

Bayesian therapeutic drug monitoring (TDM) software uses a reported pharmacokinetic (PK) model as prior information. Since its estimation is based on the Bayesian method, the estimation performance of TDM software can be improved using a PK model with characteristics similar to those of a patient. Therefore, we aimed to develop a classifier using machine learning (ML) to select a more suitable vancomycin PK model for TDM in a patient. In our study, nine vancomycin PK studies were selected, and a classifier was created to choose suitable models among them for patients. The classifier was trained using 900,000 virtual patients, and its performance was evaluated using 9000 and 4000 virtual patients for internal and external validation, respectively. The accuracy of the classifier ranged from 20.8% to 71.6% in the simulation scenarios. TDM using the ML classifier showed stable results compared with that using single models without the ML classifier. Based on these results, we have discussed further development of TDM using ML. In conclusion, we developed and evaluated a new method for selecting a PK model for TDM using ML. With more information, such as on additional PK model reporting and ML model improvement, this method can be further enhanced.

## 1. Introduction

Since its introduction into clinical practice in 1958, vancomycin has been widely used for penicillin-resistant Gram-positive bacterial infections, especially those caused by *methicillin-resistant Staphylococcus aureus* (MRSA) [1]. Adverse reactions to vancomycin typically include Red Man syndrome, nephrotoxicity, and ototoxicity. Since the adverse effects of vancomycin are related to the dosage and concentration of the drug, it can be used relatively safely under adequate monitoring [2]. Therefore, vancomycin is a representative drug for which therapeutic drug monitoring (TDM) is recommended. TDM is a clinical process that measures the concentration of a drug in the blood and interprets the resulting pharmacokinetic (PK) parameters to draw appropriate conclusions regarding drug concentration and dose adjustment [3]. A recently revised guideline recommends monitoring the area under the drug concentration-time curve (AUC) using Bayesian TDM software programs embedded with a PK model based on vancomycin data as the Bayesian prior [4].

Bayesian TDM software uses PK models reported in the existing literature as prior information, integrates patient data, and calculates patient PK parameters through statistical estimation [5]. Patient data typically include height, weight, dosing history, and drug concentration, but data on the number of blood collections in clinical practice are often limited. Hence, it is important to select an appropriate PK model to be used as prior information to correctly estimate the PK parameters from limited data. This is due to the fact that TDM performance varies depending on the PK model, even when the same patient data are used [6,7].

As vancomycin has been widely used for a long time, reported population PK studies of vancomycin for various patient groups can be used as a prior model [8]. Therefore, studies have been conducted to evaluate the predictive performance of TDM in PK models [6,7]. In particular, a study on the methods for averaging/selecting a model using goodness-of-fit has been reported [9]. The model selection and averaging approach have the advantages of reducing uncertainty that may arise from a single model assumption [9,10,11].

Machine learning (ML) has led to various breakthroughs in science and has been introduced into medicine, owing to data availability and the growth of computational power. ML is more flexible and scalable than traditional statistical methods. Thus, it has the capability of accomplishing tasks such as classification [12,13]. Although several studies have reported using ML to improve TDM performance, to the best of our knowledge, no studies have applied ML to select the appropriate PK models to be used [14,15,16].

**Figure 1:**
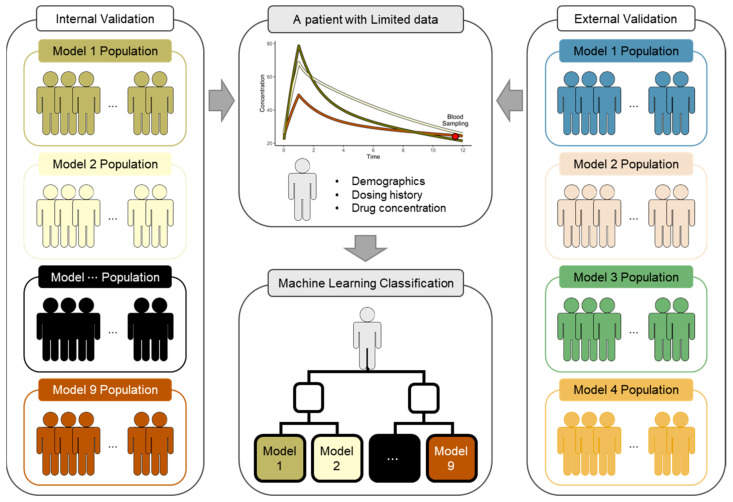
Overview of the study. The results of pharmacokinetic (PK) parameter estimation for TDM can be varied using a PK model as prior information. Therefore, the classifier was created to select a vancomycin PK model more suitable for the patient given the limited data among the nine models. The performance of TDM with the classifier applied was evaluated using the populations generated from nine PK models used as classifiers (internal validation) or from four PK models in another study (external validation).

Accordingly, the aim of this study was to develop a classifier for TDM using ML to select a vancomycin PK model appropriate for a patient (given limited data). Nine vancomycin PK studies were chosen, and a classifier to sort the patients into the PK models of those studies was created. The performance of TDM with the classifier applied was evaluated using the populations generated from nine PK models or those generated from four PK models in another study (Figure 1).

## 2. Materials and Methods

### 2.1. Classifier Development

First, PK studies were selected as labels to create classifiers. Next, virtual patients were generated as learning data using the selected PK studies, and features were created. Finally, the classifier was trained using learning data.

#### 2.1.1. PK Models for the Classifier

Nine of the fifty-four vancomycin population PK studies presented in two review articles were selected, if they met the following criteria: (1) studied population: adult; (2) compartment of PK model: two-compartment model; and (3) covariates included in the PK model: age, sex, height, weight, or renal function markers (serum creatinine [sCr], creatinine clearance [CrCL], modification of diet in renal disease [MDRD], and Chronic Kidney Disease Epidemiology Collaboration [CKD-EPI]) [17,18]. Studies in which the PK model consisted covariates that did not fall under (3) were excluded. The characteristics of the nine selected studies are listed in Table S1 [19,20,21,22,23,24,25,26,27].

#### 2.1.2. Virtual Patients for the Classifier

The demographics of 100,000 patients were generated based on representative values obtained from the internal data of the Kyung Hee University Hospital Clinical Trial Center. The mean ± SD of internal data for age (years), height (cm), weight (kg), and sCr (mg/dL) was calculated as 50.2 ± 17.1, 165.1 ± 8.7, 65.1 ± 10.2, and 0.8 ± 0.2, respectively. Sex was set as a 1:1 balance between men and women. The continuous demographic values were assumed to be from a multivariate normal distribution. The sample correlation matrix of the internal data was used for the correlation structure.

A total of 900,000 patients were generated by integrating the demographic characteristics of 100,000 patients into each of the nine selected population PK models. First, individual PK parameters were generated by integrating demographic characteristics into each population PK model with inter-individual variability. Subsequently, true concentrations were calculated from the individual PK parameters for each simulation scenario. Finally, the observed concentrations (C_OBS_) were generated by incorporating the residual unexplained variability into the true concentrations. Inter-individual variability was assumed to follow a log-normal distribution, and the residual unexplained variability was assumed to follow a normal distribution. The characteristics of the PK model are presented in Table S1.

The dosing of vancomycin was assumed to be an intravenous infusion of 1000 mg at 1-h intervals for 12 h based on the drug label provided by the Ministry of Food and Drug Safety in Korea (MFDS) [28]. The blood sampling point was set to four cases: trough (12 h); peak and trough (2, 12 h); peak, mid, and trough (2, 5, 12 h); and every hour (1, 2, 3, …, 12 h), which were applied for both single-dose and steady-states. The R package *mrgsolve* was used to generate the PK parameters and concentrations [29].

#### 2.1.3. Features and Labels

**Figure 2:**
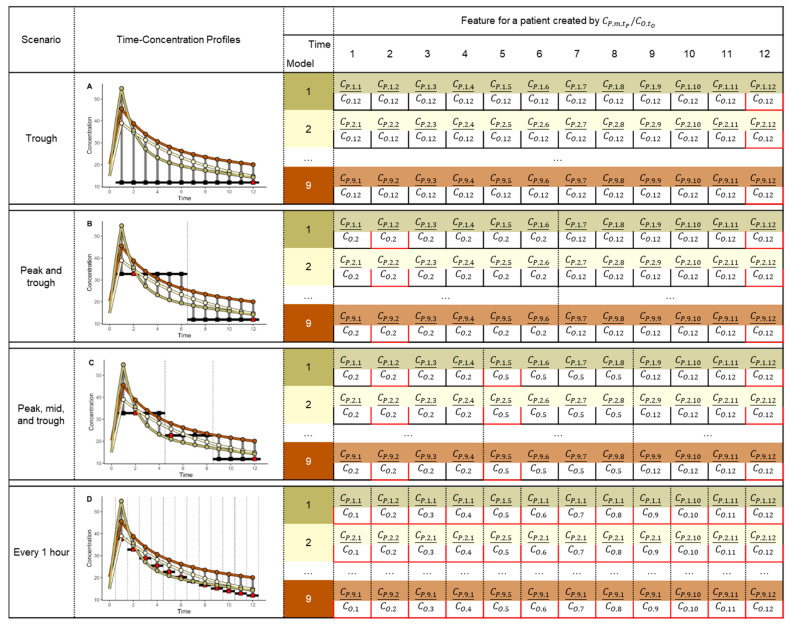
An illustrative example of feature creation. The yellow, olive, and brown lines are the time-concentration profiles of the population predicted concentration (C_PRED_) using the three models. The round dots represent the C_PRED_ of each PK model every hour for 12 h. The red squares represent the observed concentration (C_OBS_) at each blood sampling time. The black squares represent the imputed C_OBS_ at the unobserved time from the observed time. $C_{P,m,t_{P}}$ represent the C_PRED_ for a patient, where m is the number for the mth PK model of a total of nine PK models and $t_{P}$ is the time every hour from 1 to 12 h. $C_{O,t_{O}}$ represents the C_OBS_ for a patient, where $t_{O}$ is the concentration at the time observed in each blood sampling scenario. The features for classifier learning were created by dividing the C_PRED_ by the C_OBS_ for each scenario. (**A**) In the trough sampling scenario, all C_PRED_ from 1 to 12 h were divided by $C_{O,12}$; (**B**) in the peak and trough sampling scenario, the C_PRED_ from 1 to 6 h and the C_PRED_ from 7 to 12 h were divided by $C_{O,2}$ and $C_{O,12}$, respectively; (**C**) in the peak, mid, and trough sampling scenario, the C_PRED_ from 1 to 4 h, C_PRED_ from 5 to 8 h, and C_PRED_ from 9 to 12 h were divided by $C_{O,2}$, $C_{O,5}$, and $C_{O,12}$ respectively; (**D**) in the every-hour sampling scenario, the $C_{P,m,t}$ was divided by $C_{O,t}$ at each time t.

Features for classifier learning were created by dividing the population predicted concentration (C_PRED_) by the observed concentration (C_OBS_) (Figure 2). The C_PRED_ was calculated by integrating the nine PK models in Table S1 and the patient covariates (*a priori*) without incorporating any variability. The C_PRED_ could be represented as follows: $C_{PRED,m,\;t_{PRED}}^{i}$, where i is the number of the ith patient of a total of 900,000 patients, m is the number of the mth PK model of a total of nine PK models, and $t_{PRED}$ is the time for every hour from 1 to 12 h. Therefore, the C_PRED_ values were generated every hour for 12 h using the population-predicted PK parameters calculated by integrating the covariate of individual patients into each of the nine PK models; hence, a total of 108 C_PRED_ values were obtained for each patient. C_PRED_ generation was performed using the R package *mrgsolve* [29]. The observed concentrations could be represented as follows: $C_{OBS,\;t_{OBS}}^{i}$ where i is the number of the ith patient of a total of 900,000 patients and $t_{OBS}$ is the concentration observed time for each blood sampling point.

Thus, the C_PRED_ can be created every hour, but the C_OBS_ can only be known at a limited time, depending on the blood sampling time. Therefore, to match the C_PRED_ and C_OBS_ at the same time and to make the number of features equal in all scenarios, the C_OBS_ at the observed time was imputed to the C_OBS_ at the unobserved time. Thus, the C_PRED_ can be divided by C_OBS_ at the observed time and by the imputed C_OBS_ at the unobserved time. This is the same as matching and dividing C_PRED_ in a specific range of times and C_OBS_ at an observation time for each scenario. In the case of the trough sampling scenario, all $C_{PRED,m}$ for each mth PK model from 1 to 12 h were divided by $C_{OBS,12}$. For the peak and trough sampling scenarios, the $C_{PRED,m}$ from 1 to 6 h and $C_{PRED,m}$ from 7 to 12 h were divided by $C_{OBS,2}$ and $C_{OBS,12}$, respectively. In the case of the peak, mid, and trough sampling scenarios, the $C_{PRED,m}$ from 1 to 4 h, $C_{PRED,m}$ from 5 to 8 h, and $C_{PRED,m}$ from 9 to 12 h were divided by $C_{OBS,2}$, $C_{OBS,5}$, and $C_{OBS,12}$ respectively. In the case of the every-hour sampling scenario, $C_{PRED,m,t}$ was divided by $C_{OBS,t}$ at each time t.

Labels for individual patients comprised one of the nine population PK models used to generate PK parameters for each patient. Therefore, the 900,000 patients used as learning data consisted of nine groups of 100,000 patients, each with different labels. Additionally, eight different learning datasets for each scenario were generated for 900,000 patients since the composition of the features differed depending on the simulation scenario.

#### 2.1.4. Classification Model

To develop the classifiers, we first compared the prediction performances of three ML methods: Decision Tree (DT), Random Forest (RF), and XGBoost. ML models were developed using each R package as follows: (1) DT: *rpart*; (2) RF: *ranger*; and (3) XGBoost: *xgboost* [30,31,32]. The hyperparameters were then determined using 10-fold repeated cross-validation and grid search (Table S2). Since the learning data were generated based on statistical distribution, it was assumed that the characteristics of the data for hyperparameter tuning were retained even if sampled data were used. Thus, considering the computation time, 10% (*n* = 90,000) of the total learning data were randomly sampled for hyperparameter tuning. Cross-validation was applied to the training data by splitting the sampled learning data into training (70%) and test subsets (30%). Cross-validation was performed using the R package *mlr* [33]. Subsequently, the accuracies of these three models were calculated using the internal validation process described in Section 2.2. As a result, XGBoost, which had higher accuracy, was selected as the ML model for the classifier (Table 1). A classifier based on the tuned XGBoost model was used to calculate the predicted probability of each class for individual patients, which was obtained by minimizing the negative log-likelihood using the XGBoost parameter, objective (=“mult:softprob”) and eval_metric (=“mlogloss”).

### 2.2. Validation of TDM Performance

To validate the TDM performance when the classifier was applied, the AUC of vancomycin for virtual patients was predicted using a single model or an ML-selected/weighted model and compared. Therefore, new virtual patient populations were generated for validation. The PK parameters were estimated using the nine models used to generate the classifier. Subsequently, the AUC was calculated using the estimated PK parameters for each model. Additionally, the AUC was calculated from the model selected or weighed using the ML classifier. The estimated and true AUC were then compared.

#### 2.2.1. PK Models and Virtual Patients for Validation

The PK model was used to generate virtual patients for validation. Internal and external validations were performed and distinguished based on the PK model (Figure 1). The PK models for internal validation were the nine models used to develop the classifier. The PK models for external validation were the four models that did not overlap with the PK model for internal validation in the 54 vancomycin population PK studies presented in two review articles [17,18]. The PK model for external validation was selected when it met criteria (1) to (3) in Section 2.1.1. However, a PK model for external validation that also met the additional criteria of including discrete covariates, such as renal replacement therapy (RRT), was selected. The PK characteristics of the four selected studies are presented in Table S3 [34,35,36,37].

The virtual patient generation process for evaluation was the same as the patient generation process for classifier development, except for the number of patients and PK models. First, the demographic information of 1000 patients was generated using data from the Kyung Hee University Hospital Clinical Trial Center. Then, the demographics of 1000 patients were integrated into nine PK models for internal validation to generate 9000 patients and integrated into four PK models for external validation to generate 4000 patients. The vancomycin dosing and blood sampling scenarios were the same as those used for classifier development. Virtual patient generation was performed using the R package *mrgsolve* [29].

#### 2.2.2. PK Parameter Estimation

The PK parameters of the patients were estimated based on the Bayesian method, a computational combination of the patient demographics, dosing regimen, drug concentration per simulation scenario, and the PK model as prior information [5]. The PK models for estimation used the same nine models as those used to develop the classifier. Therefore, nine sets of PK parameters were obtained for each PK model for each patient. The R package *mapbayr* was used for PK parameter estimation [38].

The AUC predicted by each single model was calculated from the estimated PK parameters, giving nine values for each PK model. The AUC was calculated using the R package *mrgsolve* [29]. The time for calculating the AUC was set as the next dosing interval from the time of the concentration observation. In other words, the AUC was calculated between 12 and 24 h after the first dose.

#### 2.2.3. ML Application

For TDM performance evaluation using the ML classifier, the classifier was applied for AUC prediction in two methods: model selection and the weighted average of the models. Given the patient information for TDM, such as patient demographics, dosing regimen, and drug concentrations, the classifier calculated the probability that the patient was generated from a specific PK model among the nine label models. The model selection method picked out one PK model with the highest probability as calculated by the classifier. The AUC predicted by the selected model was used as the predicted value. The weighted average method used the probability of each PK model calculated by the classifier as the weight. The predicted AUC for each model was averaged using the weights of the corresponding models.

To compare the TDM performance using the ML classifier, two additional methods were used to predict the AUC. First, the perfect model selection method assumes that the classifier perfectly knows the PK model used to generate for individual patients (i.e., the accuracy of the classifier is 100%). In this method, the predicted AUC was the AUC predicted using the model used to generate the patient. This method was applied only to patients in the internal model, where the PK models used for patient generation were the same as those used for classifier development. Thus, it can be applied only to virtual patients for comparison purposes and not to real patients. Second, the non-weighted averaging method arithmetic averages the AUC predicted by the nine models without weights. This method was applied regardless of whether the model was internal or external. Hence, it can also be applied to both real and virtual patients.

Apart from the methods using the ML classifier, another model selection and weighted average method was applied to the evaluation data of this study [9]. In this method, the objective function values (OFVs) for estimating the PK parameters for each model were processed and used as weights. The OFVs were then calculated using the R package *mapbayr* for PK parameter estimation [38]. The OFV was processed to a weight using the following equation:

$$W_{OFV_{m}}=\frac{e^{-0.5\times OFV_{m}}}{\sum_{m=1}^{m=9}e^{-0.5\times OFV_{m}}}$$

where m is the number of the mth PK model out of the nine models used for parameter estimation. The weights of the OFVs were applied to the AUC predictions in two ways: model selection and the weighted average of the models. The PK model with the highest weight was selected, and the AUC was averaged using that weight.

#### 2.2.4. Performance Evaluation

The performance of the ML models was assessed using the metrics accuracy, precision, recall, and F1−score. These metrics are calculated as follows:

$$Accuracy=\frac{TP+TN}{TP+TN+FP+FN},\;Precision=\frac{TP}{TP+FP},\;Recall=\frac{TP}{TP+FN},\;F1-score=2\times \frac{Precision\times Recall}{Precision+Recall}$$

where TP is the number of true positives, TN is the number of true negatives, FP is the number of false positives, and FN is the number of false negatives in the confusion matrix obtained for each classification outcome of the internal validation. The confusion matrix was constructed using the R package *caret* [39].

TDM performance was assessed based on the mean percent error (MPE) and the relative root mean squared error (rRMSE) of the predicted AUC relative to the true AUC of each simulation scenario, which is defined as follows:

$$MPE=\frac{1}{N}\overset{N}{\underset{i=1}{\sum }}\frac{Predicted\;AUC_{i}-True\;AUC_{i}}{True\;AUC_{i}}\times 100\%\;rRMSE=\sqrt{\frac{1}{N}\overset{N}{\underset{i=1}{\sum }}\frac{{\left(Predicted\;AUC_{i}-True\;AUC_{i}\right)}^{2}}{{\left(True\;AUC_{i}\right)}^{2}}}\times 100\%$$

where i is the number of the ith patient of a total of N patients in each simulation scenario. For each simulation scenario, the total number of patients in the internal and external validations were 9000 and 4000, respectively. The types of predicted AUC were as follows: the AUC predicted by each model out of the nine PK models, AUC selected by the ML classifier, AUC weighted by the ML classifier, perfect selection AUC, and non-weighted averaging AUC. The true AUC was calculated from the true PK parameters generated for each patient.

## 3. Results

The classifier was created using learning data from 900,000 virtual patients based on nine PK studies and learning using the XGBoost model. The patient characteristics are presented in Table S1. The mean AUC of the learning patients by population was 178.06–290.47 mg·h/L for a single dose and 268.2–406.50 mg·h/L for the steady-state.

Table 1 lists the accuracy of the classifiers and Tables S4, S6, and S8 provide the details of the confusion matrices for each ML model by scenario. Between ML models, the XGBoost model showed the highest accuracy, and the DT model showed the lowest accuracy in all scenarios. In the XGBoost model classifier, the accuracy ranged from 24.6% to 71.6% for a single dose and 20.8% to 56.6% for the steady-state. The accuracy improved as the number of blood samples increased, and all of the ML models showed the same tendency. Similarly, in all ML models, the single-dose values were more accurate than the steady-state values. Additionally, the precision, recall, and F1-score in the ML models for each class improved as the number of observed concentrations increased (Tables S5, S7, and S9). Meanwhile, the feature importance plot of each scenario in the XGBoost model is shown in Figures S1 and S2.

For validation, the performance of TDM with the classifier was evaluated using the predicted AUC of 13,000 virtual patients (from 9000 patients in the internal validation and 4000 patients in the external validation) based on 13 PK studies. Table S1 shows the characteristics of the patients included in the internal validation. The mean AUC of the patients in the internal validation was similar to that of the patients for learning data. The characteristics of the patients in the external validation are listed in Table S3. The mean AUC of the patients in the external validation was 165.18–237.77 mg·h/L for a single dose and 317.16–691.72 mg·h/L in the steady-state.

**Figure 3:**
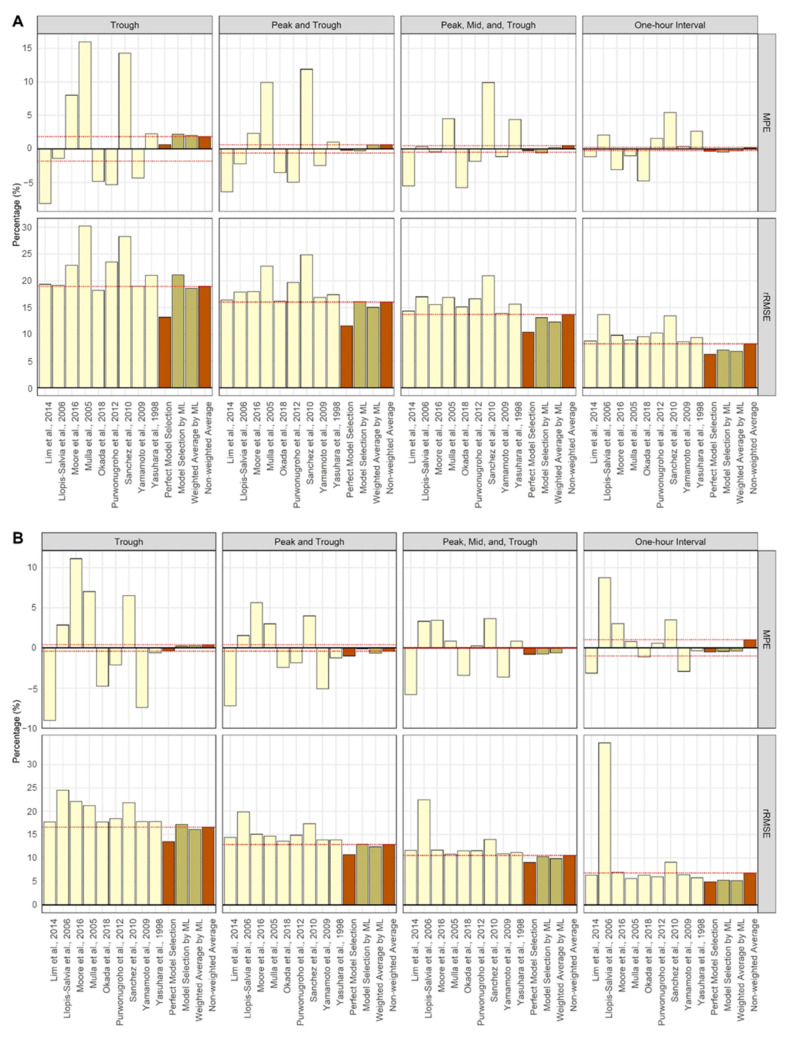
The mean percent error (MPE) and relative root mean squared error (rRMSE) of the predicted AUCs relative to the true AUCs of each simulation scenario for internal validation [19,20,21,22,23,24,25,26,27]. The prediction method (using a single model, ML application, and comparison) is distinguished by yellow, olive, and brown colors, respectively. The red dashed horizontal line is the value obtained using the non-weighted average method. (**A**) Single dose; (**B**) steady-state.

The TDM performance of the internal validation is presented in Figure 3 and Table 2. The predicted AUC of the perfect selection method showed better results for both MPE and rRMSE in most scenarios than when estimating using a single model. Except for the trough blood sampling scenario, TDM using the classifier performed better than using a single model. As the number of observed concentrations increased, the MPE and rRMSE in cases where the classifier was used approached the values of the perfect selection method. In most scenarios, the weighted average method exhibited better TDM performance than the model selection method. The non-weighted average method also showed stable results without value jumps compared to single model estimation.

**Figure 4:**
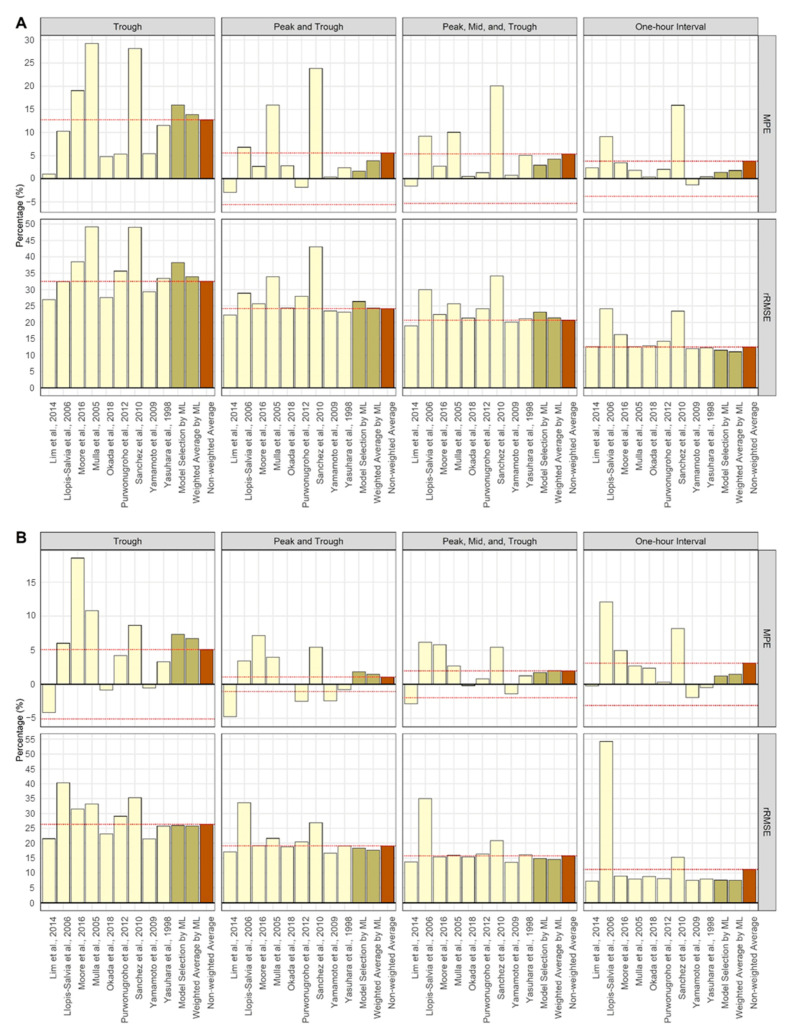
The mean percent error (MPE) and relative root mean squared error (rRMSE) of the predicted AUCs relative to the true AUCs of each simulation scenario for external validation [19,20,21,22,23,24,25,26,27]. The prediction method (using a single model, ML application, and comparison) is distinguished by yellow, olive, and brown colors, respectively. The red dashed horizontal line is the value obtained using the non-weighted average method. (**A**) Single dose; (**B**) steady-state.

The TDM performance of the external validation is shown in Figure 4 and Table 3. In the trough sampling scenario, the non-weighted average method performed better than both the model selection method and weighted average method using the ML classifier. However, as the number of observed concentrations increased, TDM performance using the classifier led to better outcomes than the non-weighted average method. The model selection method outperformed the weighted average method in terms of the MPE, but the weighted average method outperformed the model selection method in terms of the rRMSE.

Table S10 shows the TDM performance of the method using the OFVs [9]. The method using the OFV of the selection or weighted average methods showed more stable results without value jumps than a single model for patients from both internal and external validation sets. In patients in the internal validation set, the performance of TDM with the ML classifier applied was better than that of the OFV method (Table 2). In the patients in the external validation set, the method using OFV performed better than the method using the ML classifier until two concentrations were observed, but showed similar performance as the number of concentrations increased (Table 3).

## 4. Discussion

Since estimations for TDM software are mostly based on Bayesian methods, estimation performance can be improved using a PK model with PK characteristics similar to those of a patient as prior information [6,9,15]. Therefore, the purpose of classifier generation was to create a classifier that would give the patient the best TDM model (i.e., the model that most closely resembles the patient’s PK characteristics). For this purpose, the PK models used to generate the patient PK parameters were used as labels for the classifier. However, it was essential to check whether estimating the parameters with the PK model used to generate the patients could improve the performance of TDM. Therefore, as a result of testing the perfect model selection method that estimates parameters using the model used to generate the patient, it was confirmed that it showed better performance than a single model in the AUC prediction of internal validation patients (Table 2, Figure 3).

For real-world patients, there is no generation model, and the available data are limited. Therefore, the perfect model selection is impossible. Hence, we created a new feature using the ratio of C_PRED_ to C_OBS_ to enable classification is based on the available information. This is due to the fact that the trend of C_PRED_ change over time was assumed to differ by population. If so, the more similar the patient PK characteristic is to a specific PK model, the more similar the trends of change over time of the C_OBS_ (reflecting individual characteristics) may be to those of the specific C_PRED_ (reflecting population characteristics). For example, in the every-hour sampling scenario, if the patient’s characteristics were similar to the PK model used for calculating the C_PRED_, it can be assumed that the ratio of C_PRED_ to C_OBS_ would maintain a constant value every hour. The classification using these features showed high accuracy (71.6% at a single dose, every-hour sample) despite the many selection options from the nine models (Table 1). Moreover, the C_OBS_ at unobserved time points can also be used for features in our study due to the assumption that the C_PRED_ differs between populations. For example, if two patients were observed with the same trough concentration but had different covariates, the C_PRED_ cannot be identical even when using the same model. Thus, if the C_PRED_ calculated at the peak time with model X is too high for the observed trough concentration for a patient (and another patient does not), model X can be excluded from the classifications that can be used for this patient. Although the accuracy was lower than when hourly samples were used, classification was possible with 24.6% accuracy (at a single dose) even when only a trough sample was used (Table 1).

In the present study, 12 sampling points for every hour within one dosing interval were used to calculate the C_PRED_ for each model. However, an additional feature selection process may improve the classification performance [40]. The features with high importance value in our study showed that most of the time points of C_PRED_ and C_OBS_ were similar (Figures S1 and S2). In addition, the PK model has information on specific PK parameters depending on the time point [41]. Therefore, future studies will require feature selection using only the appropriate sampling points for C_PRED_ calculation.

The results of the TDM performance evaluation using the classifier were reasonable in most scenarios. However, there is one point to be considered. Between the two methods of applying ML, the weighted average method showed better MPE and rRMSE than the model selection method for both internal and external validations, except for the MPE for external validation. Patients from the external validation included special populations, such as patients with burns, continuous renal replacement therapy (CRRT), and hemodialysis (HD) [34,35,36,37]. Therefore, the AUC of the external validation set showed a different range than that of the internal model patients and had large standard deviations in some models (Tables S1 and S3). These differences in PK characteristics may have biased the results of some single-model estimates, and these biased values may be summed up when averaging the predicted AUC. Therefore, when performing TDM in patients belonging to special populations, the model selection method can be considered first. Furthermore, it is also possible to include covariates related to the special patient population during classifier generation.

In conclusion, we created and tested a classifier that could select PK models using ML and applied it to TDM to facilitate safe vancomycin administration. In general, probabilistic model selection and averaging used values related to the goodness-of-fit of the models, such as the Akaike information criterion (AIC) and Bayesian information criterion (BIC) [42]. Since the PK model is also a model for fitting data, it is possible to select a PK model based on probabilistic model selection; such a study was recently reported [9]. However, our study proposed a new method for model selection to find a model that provides better TDM performance with limited patient data, such as sex, age, height, weight, and concentration, without model fitting.

In the era of big data, our research method with ML-based classification is expected to further develop as the amount of available information increases. The results of our study showed that regardless of internal and external validation, increasing the number of observed concentrations resulted in better classification accuracy (Table 1). In particular, internal validation showed better TDM performance than the previously reported method (using OFVs) in almost all scenarios (Table 2 and  Table S10). To assume the clinical practice where limited patient information is available, we only selected nine PK models with easily measurable covariates for classifier generation in our study. Theoretically, if all PK models were built for all vancomycin patient populations and were included in a classifier, TDM with the classifier applied will always achieve better performance than TDM using a single model. Additionally, if additional covariates not used in our study were used to generate a classifier, a classifier with more information can be created. Furthermore, our ML-based approach can be easily applied to the TDM of any drug using the same procedure as creating the classifier for vancomycin, particularly if various PK models can be used as prior information.

It was also observed that the classification performance improved when the improved algorithm, XGBoost, was used since it is an optimized distributed gradient boosting library designed to be more efficient and scalable than traditional classification models, such as DT (Table 1) [43]. Since the main purpose of this study was not to improve the performance of classification, only three ML models were used and compared. Therefore, future studies may also consider using a super-runner model to improve classification performance. In addition, computing and ML techniques are developing rapidly, and these advances can help improve the selection of ML-based TDM models.

Although we developed a new method for TDM model selection and evaluated the method, it had certain limitations. Currently, it applies only to scenarios in which the classifier is trained in advance. Therefore, further studies are needed to apply our method to commercial TDM programs. For example, depending on the hospital, generating classifiers by learning only frequently used scenarios in advance may be considered. To apply a general multiple dosing regimen, it is also possible to create a classifier using patient data that changes when the renal function changes over time, considering the PK characteristics of vancomycin excreted by the kidneys. Moreover, it is possible to develop a new set of features that can classify the PK model regardless of the scenario. Another method is to find an appropriate amount of training data and features to speed up computation, creating a new classifier for a new patient every time. Currently, it takes approximately 2 min to create one XGBoost classifier with data from 900,000 virtual patients using a 64-bit Windows 11 platform with an Intel i7-9700 CPU, 16 GB RAM, and NVIDIA TITAN Xp with 12 GB VRAM. As an additional limitation, all processes in this study were only based on simulations. The entire process was conducted based on simulations, and the obtained values were compared with the true values in various scenarios. Nevertheless, to make the values similar to real-world patients, demographic information was generated using the internal data. However, further studies are required to validate the performance of TDM with an ML classifier applied to real patients. Further studies overcoming these limitations can help improve the TDM performance for safe vancomycin administration.

## 5. Conclusions

In this study, we created and tested a classifier that selects PK models using ML and applied it to TDM to ensure safe vancomycin administration. The accuracy of the classifier ranged from 20.8 to 71.6% in various simulation scenarios. The TDM performance using the ML classifier showed stable results compared with using single models. In the era of big data, this new method for TDM model selection will develop further as the amount of available information increases.

## Figures and Tables

**Table 1:** The accuracy of the ML models in each scenario.

Scenarios	Trough (%)	Peak and Trough (%)	Peak, Mid, and Trough (%)	One-HourInterval (%)
Decision Tree				
Single Dose	21.0	22.2	30.5	31.1
Steady State	16.8	20.7	22.9	27.0
Random Forest				
Single Dose	23.4	30.7	42.6	68.6
Steady State	19.1	27.0	33.3	54.4
XGBoost				
Single Dose	24.6	31.8	42.7	71.6
Steady State	20.8	27.8	33.7	56.6

**Table 2:** The mean percent error (MPE) and relative root mean squared error (rRMSE) of the predicted AUC relative to the true AUC of each simulation scenario for internal validation.

Measures	MPE (%)				rRMSE (%)			
Scenarios	Trough	Peak and Trough	Peak, Mid, and Trough	One-Hour Interval	Trough	Peak and Trough	Peak, Mid, and Trough	One-Hour Interval
**Single Dose Model**								
Lim et al., 2014 [19]	−8.16	−6.40	−5.50	−1.15	19.36	16.40	14.36	8.75
Llopis-Salvia et al., 2006 [20]	−1.39	−2.24	0.32	2.10	19.18	17.92	17.03	13.70
Moore et al., 2016 [21]	8.02	2.31	−0.44	−3.11	22.93	17.98	15.58	9.83
Mulla et al., 2005 [22]	15.97	9.91	4.49	−1.02	30.25	22.75	16.93	8.97
Okada et al., 2018 [23]	−4.86	−3.53	−5.76	−4.79	18.25	16.23	15.14	9.56
Purwonugroho et al., 2012 [24]	−5.33	−4.96	−1.86	1.59	23.52	19.72	16.63	10.25
Sánchez et al., 2010 [25]	14.31	11.88	9.90	5.42	28.29	24.89	20.94	13.46
Yamamoto et al., 2009 [26]	−4.32	−2.45	−1.13	0.38	19.03	16.89	13.94	8.64
Yasuhara et al., 1998 [27]	2.27	1.01	4.40	2.64	21.05	17.42	15.64	9.39
Perfect Model Selection	0.65	−0.23	−0.26	−0.35	13.19	11.62	10.44	6.25
Model Selection by ML	2.22	−0.31	−0.60	−0.46	21.10	16.15	13.12	7.09
Weighted Average by ML	2.00	0.59	0.19	−0.27	18.60	15.07	12.32	6.84
Non-weighted average	1.83	0.61	0.49	0.23	18.97	16.04	13.71	8.22
**Steady-State Model**								
Lim et al., 2014 [19]	−9.02	−7.21	−5.78	−3.13	17.70	14.40	11.60	6.33
Llopis-Salvia et al., 2006 [20]	2.85	1.55	3.32	8.73	24.55	19.89	22.46	34.66
Moore et al., 2016 [21]	11.12	5.65	3.45	3.02	22.06	15.08	11.68	7.00
Mulla et al., 2005 [22]	7.02	3.00	0.87	0.82	21.23	14.63	10.80	5.63
Okada et al., 2018 [23]	−4.74	−2.40	−3.39	−1.14	17.73	13.57	11.55	6.35
Purwonugroho et al., 2012 [24]	−2.11	−1.84	0.26	0.60	18.45	14.89	11.57	6.00
Sánchez et al., 2010 [25]	6.52	4.00	3.64	3.48	21.83	17.32	13.98	9.08
Yamamoto et al., 2009 [26]	−7.40	−5.06	−3.62	−2.91	17.78	13.87	10.92	6.43
Yasuhara et al., 1998 [27]	−0.59	−1.25	0.84	−0.36	17.81	13.86	11.19	5.78
Perfect Model Selection	−0.35	−0.99	−0.79	−0.48	13.51	10.76	9.07	4.94
Model Selection by ML	0.25	−0.11	−0.77	−0.42	17.17	12.95	10.28	5.28
Weighted Average by ML	0.27	−0.64	−0.61	−0.40	16.11	12.40	9.87	5.18
Non-weighted Average	0.41	−0.39	−0.05	1.01	16.59	12.87	10.56	6.80

**Table 3:** The mean percent error (MPE) and relative root mean squared error (rRMSE) of the predicted AUC relative to the true AUC of each simulation scenario for external validation.

Measures	MPE (%)				rRMSE (%)			
Scenarios	Trough	Peak and Trough	Peak, Mid and Trough	One-hour Interval	Trough	Peak and Trough	Peak, Mid and Trough	One-hour Interval
**Single Dose Model**								
Lim et al., 2014 [19]	1.03	−2.92	−1.56	2.35	26.98	22.23	18.97	12.62
Llopis-Salvia et al., 2006 [20]	10.26	6.81	9.22	9.15	32.41	28.91	29.97	24.11
Moore et al., 2016 [21]	19.04	2.69	2.73	3.41	38.48	25.67	22.43	16.30
Mulla et al., 2005 [22]	29.24	15.97	10.05	1.81	49.13	33.94	25.69	12.62
Okada et al., 2018 [23]	4.77	2.78	0.52	0.40	27.58	24.37	21.31	12.88
Purwonugroho et al., 2012 [24]	5.31	−1.82	1.32	2.05	35.64	27.92	24.13	14.28
Sánchez et al., 2010 [25]	28.13	23.84	20.07	15.89	48.98	43.01	34.17	23.44
Yamamoto et al., 2009 [26]	5.42	0.42	0.77	−1.33	29.37	23.46	20.07	11.96
Yasuhara et al., 1998 [27]	11.54	2.40	5.07	0.43	33.43	23.16	21.08	12.22
Model Selection by ML	15.91	1.65	2.95	1.37	38.21	26.37	23.11	11.53
Weighted Average by ML	13.90	3.89	4.27	1.78	33.91	24.34	21.41	11.04
Non-weighted average	12.75	5.58	5.36	3.79	32.49	24.20	20.67	12.49
**Steady-State Model**								
Lim et al., 2014 [19]	−4.15	−4.73	−2.86	−0.25	21.48	17.13	13.76	7.28
Llopis-Salvia et al., 2006 [20]	6.02	3.43	6.16	12.11	40.31	33.66	35.01	54.27
Moore et al., 2016 [21]	18.54	7.16	5.82	4.97	31.51	19.18	15.40	8.94
Mulla et al., 2005 [22]	10.82	3.97	2.67	2.69	33.25	21.63	15.89	7.97
Okada et al., 2018 [23]	−0.85	−0.05	−0.20	2.37	23.11	18.78	15.38	8.83
Purwonugroho et al., 2012 [24]	4.21	−2.49	0.79	0.32	29.06	20.40	16.35	8.11
Sánchez et al., 2010 [25]	8.66	5.46	5.43	8.17	35.34	26.83	20.84	15.27
Yamamoto et al., 2009 [26]	−0.56	−2.45	−1.41	−1.94	21.37	16.68	13.64	7.59
Yasuhara et al., 1998 [27]	3.28	−0.77	1.26	−0.49	25.72	19.08	16.15	7.98
Model Selection by ML	7.32	1.84	1.73	1.21	25.97	18.34	14.83	7.62
Weighted Average by ML	6.74	1.48	2.02	1.46	25.80	17.68	14.56	7.61
Non-weighted Average	5.11	1.06	1.96	3.11	26.34	19.06	15.77	11.20

## Data Availability

Data will be made available on request.

## Supplementary Materials

The following supporting information can be downloaded at: https://www.mdpi.com/article/10.3390/pharmaceutics14051023/s1, Table S1. PK model and patient characteristics used for classifier training and internal validation. Table S2. Hyperparameter ranges used for tuning the machine learning (ML) models. Table S3. PK model and patient characteristics in the external validation set. Table S4. The confusion matrix of the decision tree (DT) model in each scenario. Table S5. The precision, recall, and F1-Score of the decision tree (DT) model in each scenario. Table S6. The confusion matrix of the random forest (RF) model in each scenario. Table S7. The precision, recall, and F1-Score of the random forest (RF) model in each scenario. Table S8. The confusion matrix of the XGBoost model in each scenario. Table S9. The precision, recall, and F1-Score of XGBoost in each scenario. Table S10. The mean percent error (MPE) and relative root mean squared error (rRMSE) of the predicted AUC relative to the true AUC of each simulation scenario using objective function values (OFVs) for model selection and weighted averaging. Figure S1. The feature importance plot of the XGBoost model in a single dose. The x-axis represents the XGBoost importance value of the feature, whereas the y-axis represents the concentration used for feature creation. Out of the 108 features created, 10 features with the highest importance values are presented. (A) Trough, (B) peak and trough, (C) peak, mid, and trough, and (D) one-hour interval sampling. Figure S2. The feature importance plot of the XGBoost model in the steady state. The x-axis represents the XGBoost importance value of the feature, whereas the y-axis represents the concentration used for feature creation. Out of the 108 features created, 10 features with the highest importance values are presented. (A) Trough, (B) peak and trough, (C) peak, mid, and trough, and (D) one-hour interval sampling.
