# Supplementary material for: Application of Machine Learning Classification to Improve the Performance of Vancomycin Therapeutic Drug Monitoring
Source: Pharmaceutics. 2022 May 9;14(5):1023. doi: 10.3390/pharmaceutics14051023 (PMC9144093; doi:10.3390/pharmaceutics14051023)
Supplement: Supplementary file 1 [file pharmaceutics-14-01023-s001.zip › pharmaceutics-1677636-supplementary.pdf]

Table S1. PK model and patient characteristics used for classifier training and internal validation.

Reference	Patient population	Parameters	Typical Value	IIV	RUV	Patients for Classifier Learning (Mean ± SD)	Patients for Internal Validation (Mean ± SD)
Lim *et al.*, 2014	Infection with MRSA	CL	3.96×($CLCR_{BW}$/100)	40.1%	P: 0.231 mg/L	4.21 ± 2.16	4.31 ± 2.26
		V_c	33.1	35.7%		34.69 ± 10.62	34.6 ± 10.48
		V_p	48.3	-		48.3 ± 0	48.3 ± 0
		Q	7.48	71.8%		8.18 ± 4.75	8.02 ± 4.74
		$AUC_{Single\ Dose}$				178.06 ± 46.45	176.36 ± 46.58
		$AUC_{Steady\ State}$				301.55 ± 154.68	297.21 ± 149.42
Llopis-Salvia *et al.*, 2006	critically-ill patients	CL	0.034×$CLCR_{LBW}$ + 0.015×WT	29.2%	P: 23.9%<br>A: 18.5%	3.42 ± 1.44	3.47 ± 1.47
		V_c	0.414×WT	36.4%		25.89 ± 9.29	25.8 ± 9.43
		V_p	1.32×WT	39.8%		82.73 ± 31.64	82.77 ± 32.66
		Q	7.48	-		7.48 ± 0	7.48 ± 0
		$AUC_{Single\ Dose}$				180.74 ± 50.47	180.43 ± 52.05
		$AUC_{Steady\ State}$				346.79 ± 152.72	341.91 ± 151.09
Moore *et al.*, 2016	ECMO patients	CL	2.83×(1+0.0154×($CLCR_{BW}$-83))	77%	P: σ^2 =0.0067	4.31 ± 3.48	4.45 ± 3.55
		V_c	24.2×(1+0.0638×(WT-94.5))	34%		20.15 ± 6.17	20.1 ± 6.14
		V_p	32.3×(1+0.0169×(WT-94.5))	-		14.88 ± 5.86	15.26 ± 5.86
		Q	11.2	-		11.2 ± 0	11.2 ± 0
		$AUC_{Single\ Dose}$				290.47 ± 154.62	286.08 ± 158.25
		$AUC_{Steady\ State}$				406.5 ± 349.98	399.88 ± 327.73
Mulla *et al.*, 2005	ECMO patients	CL	(4.3/SCr)×WT	25%	P: 12.1%<br>A: 2.1 mg/L	4.07 ± 1.48	4.08 ± 1.48
		V_c	0.47×WT	25%		23.7 ± 6.61	24.27 ± 6.87
		V_p	0.25×WT	48%		16.93 ± 7.52	17.29 ± 7.39
		Q	0.09×WT	91%		7.08 ± 5.2	7.01 ± 5.37
		$AUC_{Single\ Dose}$				236.53 ± 69.45	234.16 ± 68.32
		$AUC_{Steady\ State}$				278.93 ± 101.36	274.44 ± 101.04
Okada *et al.*, 2018	patients undergoing allogeneic hematopoietic stem-cell	CL	4.25×($CLCR_{BSA\ adj.}$/113)$^{0.7}$	25.2%	P: 17.2%	4.07 ± 1.25	4.11 ± 1.26
		V_c	39.2×(WT/59.4)$^{0.787}$	14.2%		41.04 ± 7.5	41.6 ± 7.59
		V_p	56.1	66.9%		64.63 ± 35.2	64.51 ± 35.45
		Q	1.95	-		1.95 ± 0	1.95 ± 0
		$AUC_{Single\ Dose}$				184.27 ± 36.18	183.09 ± 36.66
		$AUC_{Steady\ State}$				268.61 ± 82.84	268.18 ± 83.41
		CL	0.044×$CLCR_{BW}$	35.78%	A: 4.51 mg/L	4.64 ± 2.26	4.67 ± 2.17

Purwonugroho *et al.*, 2012	hospitalized patients at any ward	V_c	0.542×Age	20.93%		24.8 ± 8.85	24.36 ± 8.46
		V_p	44.2	57.27%		49.33 ± 23.45	49.34 ± 23.02
		Q	6.95	39.5%		7.33 ± 2.47	7.47 ± 2.62
		$AUC_{Single\ Dose}$				179.14 ± 53.22	178.16 ± 52.81
		$AUC_{Steady\ State}$				268.28 ± 131.56	264.01 ± 133.11
Sánchez *et al.*, 2010	hospitalized patients	CL	$0.157+0.563\times CLCR_{BW}$	24.49%	P: 24.9%	3.63 ± 1.49	3.73 ± 1.55
		V_c	$0.283 \times WT$	-		17.71 ± 3.04	17.91 ± 3.04
		V_p	$32.2 \times (Age/53.5)$	6.8%		27.13 ± 8.35	27.08 ± 8.34
		Q	$0.111 \times WT$	-		6.95 ± 1.19	7.03 ± 1.19
		$AUC_{Single\ Dose}$				244.01 ± 63.13	239.92 ± 62.26
		$AUC_{Steady\ State}$				322.13 ± 131.43	319.04 ± 132.44
Yamamoto *et al.*, 2009	patients suffering from gram positive infections	CL	3.83, if $CLCR_{BW} \geq 85$ mL/min<br>$0.32\times CLCR_{BW} + 0.32$, if $CLCR_{BW} < 85$ mL/min	37.5%	E: 14.3%	3.48 ± 1.36	3.56 ± 1.38
		V_c	$0.478 \times WT$	18.2%		30.3 ± 7.14	30.44 ± 7.27
		V_p	60.6	72.8%		71.27 ± 41.82	72.11 ± 42.05
		Q	8.81	19.2%		8.94 ± 1.5	8.92 ± 1.5
		$AUC_{Single\ Dose}$				182.15 ± 49.14	179.3 ± 46.94
		$AUC_{Steady\ State}$				336.06 ± 144.12	335.14 ± 140.28
Yasuhara *et al.*, 1998	Infection with MRSA	CL	3.51, if $CLCR_{BW} > 85$ mL/min<br>$0.0478\times CLCR_{BW}$, if $CLCR_{BW} \leq 85$ mL/min	38.5%	E: 23.7%	3.59 ± 1.25	3.64 ± 1.23
		V_{ss}	60.7	25.4%		18.09 ± 5.13	62.03 ± 13.53
		k_{12}	0.525	-		44.09 ± 10.28	0.52 ± 0
		k_{21}	0.213	28.6%		9.5 ± 2.69	0.22 ± 0.05
		$AUC_{Single\ Dose}$				213.51 ± 48.28	211.36 ± 46.74
		$AUC_{Steady\ State}$				315.42 ± 115.94	315.01 ± 117.39

Note: The number of patients for classifier learning and evaluation was 900,000 and 9,000, respectively. Abbreviations: CL, clearance; V_c, central volume of distribution; V_P, peripheral volume of distribution; Q, intercompartmental clearance; V_{ss}, volume of distribution at steady-state; k_{12}, first-order transfer rate constant from the central compartment to the peripheral compartment; k_{21}, first-order transfer rate; $CLCR_{BW}$, creatinine clearance using total body weight; sCr, serum creatinine; $CLCR_{BSA\ adj.}$, creatinine clearance using the body weight adjusted by the body surface area; WT, total body weight; IIV, interindividual variability; RUV, residual unexplained variability; P, proportional; A, additive; E, exponential component; AUC, area under the drug concentration-time curve.

Table S2. Hyperparameter ranges used for tuning the machine learning (ML) models.

Model	Parameter	Range
**Decision Tree**	minsplit	5, 6, 7, 8, 9, 10
	minbucket	2, 3
	cp	0.01, 0.05
**Random Forest**	mtry	1, 5, 10
	splitrule	gini, extratrees
	min.node.size	10, 20
**XGBoost**	eta	0.025, 0.05, 0.1, 0.3
	max_depth	2, 3, 4, 5, 6
	subsample	0.5, 0.75, 1
	colsample_bytree	0.5, 0.75, 1
	nrounds	Every 50 from 200 to 1000

Table S3. PK model and patient characteristics in the external validation set.

Reference	Patient population	Parameters	Typical Value	IIV**	RUV**	Patients for External Validation (Mean ± SD)
Bae *et al.*, 2019	hospitalized patients	CL	$2.82\times(CLCR_{BW}/72)^{0.836}$	40.1%	P: 0.253 mg/L	4.21 ± 3.78
		V_c	31.8	35.7%		31.8 ± 0
		V_p	$75.4\times(WT/70)$	-		85.69 ± 38.43
		Q	11.7	71.8%		11.7 ± 0
		$AUC_{Single\ Dose}$				165.18 ± 62.42
		$AUC_{Steady\ State}$				691.72 ± 1190.7
Dolton *et al.*, 2010	patients with severe burn injuries	CL	$4.7\times(CLCR_{LBW}/6.53)$	32.7%	P: 29.3%<br>A: 0.2292 mg/L	3.35 ± 1.7
		V_c	$68.4\times(WT/70) - 31.8\times BUN$	19.1%		44.63 ± 21.72
		V_p	$75.4\times(WT/70)$	172.6%		107.54 ± 116.8
		Q	4.54	-		4.54 ± 0
		$AUC_{Single\ Dose}$				191.23 ± 76.33
		$AUC_{Steady\ State}$				381.52 ± 197.16
Goti *et al.*, 2018	hospitalized patients with high prevalence of end-stage renal disease	CL	$4.5\times(CLCR_{BW}/120)^{0.8}\times0.7^{DIAL}$	39.8%	P: 22.7%<br>A: 3.4 mg/L	3.8 ± 1.83
		V_c	$58.4\times(WT/70)\times0.5^{DIAL}$	81.6%		58.57 ± 42.9
		V_p	38.4	57.1%		42.87 ± 20.32
		Q	6.5	-		6.5 ± 0
		$AUC_{Single\ Dose}$				177.6 ± 65.62
		$AUC_{Steady\ State}$				317.16 ± 155.94
Medellín-Garibay *et al.*, 2016	trauma patients	CL	$0.49\times CLCR_{BW}$,<br>$0.34\times CLCR_{BW}$, if furosemide	37.0%	P: 19.2%<br>A: 3.5 mg/L	2.9 ± 1.51
		V_c	$1.07\times WT$, if Age > 65 years<br>$0.74\times WT$, if Age ≤ 65 years	40.0%		51.13 ± 20.08
		V_p	$5.9\times WT$	-		373.4 ± 63.32
		Q	0.81	-		0.81 ± 0
		$AUC_{Single\ Dose}$				237.77 ± 72.79
		$AUC_{Steady\ State}$				436.31 ± 236.19

Note: The number of evaluated patients was 4,000. Abbreviations: CL, clearance; V_c, central volume of distribution; V_P, peripheral volume of distribution; Q, intercompartmental clearance; $CLCR_{BW}$, creatinine clearance using total body weight; $CLCR_{LBW}$, creatinine clearance using lean body weight; WT, total body weight; BUN, a dichotomous covariate coded as BUN = 0 if the patients had burns; DIAL, a dichotomous covariate coded as DIAL = 0 if the patients were undergoing hemodialysis; IIV, interindividual variability; RUV, residual unexplained variability; P, proportional; A, additive component; AUC, area under the drug concentration-time curve.

Table S4. The confusion matrix of the decision tree (DT) model in each scenario.

	Actual Class (%)	Lim et al., 2014	Llopis-Salvia et al., 2006	Moore et al., 2016	Mulla et al., 2005	Okada et al., 2018	Purwonugroho et al., 2012	Sanchez et al., 2010	Yamamoto et al., 2009	Yasuhara et al., 1998
**Trough Sampling at Single Dose**										
Predicted Class	Lim et al., 2014	0.0	0.0	0.0	0.0	0.0	0.0	0.0	0.0	0.0
	Llopis-Salvia et al., 2006	7.8	9.0	3.2	4.0	7.6	4.3	4.8	7.7	5.8
	Moore et al., 2016	0.0	0.2	3.8	1.0	0.0	0.8	0.5	0.1	0.5
	Mulla et al., 2005	0.0	0.0	0.0	0.0	0.0	0.0	0.0	0.0	0.0
	Okada et al., 2018	0.0	0.0	0.0	0.0	0.0	0.0	0.0	0.0	0.0
	Purwonugroho et al., 2012	0.3	0.1	0.9	0.6	0.0	2.4	0.0	0.1	0.0
	Sánchez et al., 2010	3.1	1.9	3.2	5.6	3.5	3.7	5.8	3.2	4.8
	Yamamoto et al., 2009	0.0	0.0	0.0	0.0	0.0	0.0	0.0	0.0	0.0
	Yasuhara et al., 1998	0.0	0.0	0.0	0.0	0.0	0.0	0.0	0.0	0.0
**Trough Sampling at Steady-State**										
Predicted Class	Lim et al., 2014	0.0	0.0	0.0	0.0	0.0	0.0	0.0	0.0	0.0
	Llopis-Salvia et al., 2006	0.0	0.0	0.0	0.0	0.0	0.0	0.0	0.0	0.0
	Moore et al., 2016	0.3	0.0	2.2	0.9	0.0	1.6	0.1	0.0	0.2
	Mulla et al., 2005	0.0	0.0	0.0	0.0	0.0	0.0	0.0	0.0	0.0
	Okada et al., 2018	8.1	7.5	5.2	9.4	10.4	8.0	9.3	6.9	7.9
	Purwonugroho et al., 2012	0.0	0.0	0.0	0.0	0.0	0.0	0.0	0.0	0.0
	Sánchez et al., 2010	0.0	0.0	0.0	0.0	0.0	0.0	0.0	0.0	0.0
	Yamamoto et al., 2009	2.7	3.6	3.7	0.8	0.7	1.5	1.7	4.3	3.0
	Yasuhara et al., 1998	0.0	0.0	0.0	0.0	0.0	0.0	0.0	0.0	0.0
**Peak and Trough Sampling at Single Dose**										
Predicted Class	Lim et al., 2014	2.7	0.8	0.3	0.1	0.5	1.1	0.1	0.4	0.7
	Llopis-Salvia et al., 2006	0.0	0.0	0.0	0.0	0.0	0.0	0.0	0.0	0.0
	Moore et al., 2016	0.0	0.0	2.8	0.3	0.0	0.2	0.2	0.0	0.3
	Mulla et al., 2005	0.0	0.0	0.0	0.0	0.0	0.0	0.0	0.0	0.0
	Okada et al., 2018	0.0	0.0	0.0	0.0	0.0	0.0	0.0	0.0	0.0
	Purwonugroho et al., 2012	0.0	0.0	0.0	0.0	0.0	0.0	0.0	0.0	0.0
	Sánchez et al., 2010	2.2	3.7	3.5	6.3	1.5	3.2	7.2	1.1	4.5
	Yamamoto et al., 2009	6.2	6.6	4.6	4.4	9.1	6.6	3.7	9.6	5.6
	Yasuhara et al., 1998	0.0	0.0	0.0	0.0	0.0	0.0	0.0	0.0	0.0
**Peak and Trough Sampling at Steady-State**										
P	Lim et al., 2014	0.0	0.0	0.0	0.0	0.0	0.0	0.0	0.0	0.0

	**Llopis-Salvia et al., 2006**	0.0	0.0	0.0	0.0	0.0	0.0	0.0	0.0	0.0
	**Moore et al., 2016**	0.6	0.7	4.1	1.0	0.0	1.7	0.4	0.3	0.9
	**Mulla et al., 2005**	0.0	0.0	0.0	0.0	0.0	0.0	0.0	0.0	0.0
	**Okada et al., 2018**	6.8	5.4	3.8	7.1	9.2	6.9	5.8	6.0	5.6
	**Purwonugroho et al., 2012**	0.0	0.0	0.0	0.0	0.0	0.0	0.0	0.0	0.0
	**Sánchez et al., 2010**	1.3	2.1	1.5	2.4	1.1	1.1	3.4	0.8	2.3
	**Yamamoto et al., 2009**	2.4	2.9	1.7	0.7	0.7	1.4	1.4	3.9	2.4
	**Yasuhara et al., 1998**	0.0	0.0	0.0	0.0	0.0	0.0	0.0	0.0	0.0
**Peak, Mid, and Trough Sampling at Single Dose**										
**Predicted Class**	**Lim et al., 2014**	2.5	0.6	0.0	0.1	0.3	0.7	0.1	0.4	0.7
	**Llopis-Salvia et al., 2006**	0.7	2.1	0.1	0.8	0.1	1.0	0.7	0.3	1.0
	**Moore et al., 2016**	0.4	0.4	4.6	0.7	0.0	1.7	0.2	0.3	0.5
	**Mulla et al., 2005**	1.0	0.4	3.2	5.2	1.3	1.2	4.1	0.4	1.5
	**Okada et al., 2018**	1.2	0.8	1.3	2.3	5.1	1.6	1.3	1.1	0.7
	**Purwonugroho et al., 2012**	0.0	0.0	0.0	0.0	0.0	0.4	0.1	0.0	0.1
	**Sánchez et al., 2010**	0.5	1.2	0.1	0.6	0.3	1.0	2.6	0.5	2.1
	**Yamamoto et al., 2009**	4.8	5.6	1.9	1.4	3.9	3.5	2.0	8.2	4.5
	**Yasuhara et al., 1998**	0.0	0.0	0.0	0.0	0.0	0.0	0.0	0.0	0.0
**Peak, Mid, and Trough Sampling at Steady-State**										
**Predicted Class**	**Lim et al., 2014**	0.0	0.0	0.0	0.0	0.0	0.0	0.0	0.0	0.0
	**Llopis-Salvia et al., 2006**	0.0	0.0	0.0	0.0	0.0	0.0	0.0	0.0	0.0
	**Moore et al., 2016**	0.8	0.5	4.4	1.1	0.1	1.8	0.4	0.3	0.5
	**Mulla et al., 2005**	0.0	0.0	0.0	0.0	0.0	0.0	0.0	0.0	0.0
	**Okada et al., 2018**	4.3	3.7	2.9	5.7	8.0	3.8	4.2	4.2	3.4
	**Purwonugroho et al., 2012**	1.7	0.9	0.4	0.8	0.4	2.3	0.8	1.0	1.7
	**Sánchez et al., 2010**	1.0	1.8	1.1	2.2	0.9	0.9	3.1	0.6	1.9
	**Yamamoto et al., 2009**	3.3	4.1	2.3	1.3	1.7	2.3	2.5	5.1	3.7
	**Yasuhara et al., 1998**	0.0	0.0	0.0	0.0	0.0	0.0	0.0	0.0	0.0
**One-hour Interval Sampling at Single Dose**										
**Predicted Class**	**Lim et al., 2014**	2.0	0.5	0.0	0.0	0.6	0.4	0.0	0.3	0.1
	**Llopis-Salvia et al., 2006**	0.0	0.0	0.0	0.0	0.0	0.0	0.0	0.0	0.0
	**Moore et al., 2016**	0.0	0.0	3.3	0.8	0.0	0.0	0.3	0.0	0.3
	**Mulla et al., 2005**	0.5	0.3	2.8	4.2	0.4	1.2	3.4	0.3	1.0
	**Okada et al., 2018**	2.6	1.3	1.4	2.1	8.4	1.7	0.5	2.0	0.5
	**Purwonugroho et al., 2012**	0.0	0.0	0.0	0.0	0.0	0.0	0.0	0.0	0.0
	**Sánchez et al., 2010**	0.0	0.0	0.0	0.0	0.0	0.0	0.0	0.0	0.0

	**Yamamoto et al., 2009**	3.1	2.9	0.6	0.6	1.3	2.0	0.4	5.4	1.4
	**Yasuhara et al., 1998**	2.8	6.0	3.0	3.3	0.3	5.8	6.5	3.2	7.8
**One-hour Interval Sampling at Steady-State**										
**Predicted Class**	**Lim et al., 2014**	0.0	0.0	0.0	0.0	0.0	0.0	0.0	0.0	0.0
	**Llopis-Salvia et al., 2006**	0.0	0.0	0.0	0.0	0.0	0.0	0.0	0.0	0.0
	**Moore et al., 2016**	0.7	0.5	3.7	0.7	0.1	0.9	0.2	0.3	0.4
	**Mulla et al., 2005**	1.1	1.7	2.3	4.6	0.6	2.2	3.5	1.2	1.7
	**Okada et al., 2018**	6.3	3.8	1.6	4.0	9.4	3.9	1.9	4.7	2.3
	**Purwonugroho et al., 2012**	0.5	0.6	0.7	0.3	0.0	1.9	1.2	0.3	1.2
	**Sánchez et al., 2010**	0.0	0.0	0.0	0.0	0.0	0.0	0.0	0.0	0.0
	**Yamamoto et al., 2009**	1.9	2.5	1.5	1.1	0.9	1.7	1.5	3.8	1.8
	**Yasuhara et al., 1998**	0.6	2.1	1.3	0.3	0.0	0.6	2.9	0.7	3.6

Table S5. The precision, recall, and F1-Score of the decision tree (DT) model in each scenario.

Sampling Scenarios	Trough (%)			Peak and Trough (%)			Peak, Mid, and Trough (%)			One-hour Interval (%)		
**Measures**	**Preci-sion**	**Recall**	**F1-Score**	**Preci-sion**	**Recall**	**F1-Score**	**Preci-sion**	**Recall**	**F1-Score**	**Preci-sion**	**Recall**	**F1-Score**
**Class in the Single Dose**												
Lim et al., 2014	-	0.0	-	40.0	24.3	30.2	46.2	22.6	30.4	51.4	18.4	27.1
Llopis-Salvia et al., 2006	16.6	81.0	27.6	-	0.0	-	29.9	18.5	22.9	-	0.0	-
Moore et al., 2016	55.4	33.8	42.0	72.1	24.8	36.9	52.0	41.0	45.9	68.9	30.1	41.9
Mulla et al., 2005	-	0.0	-	-	0.0	-	28.4	46.7	35.3	29.5	37.5	33.0
Okada et al., 2018	-	0.0	-	-	0.0	-	33.2	45.9	38.6	40.6	75.6	52.9
Purwonugroho et al., 2012	55.0	21.4	30.8	-	0.0	-	56.1	3.2	6.1	50.0	0.3	0.6
Sánchez et al., 2010	16.8	52.5	25.4	21.6	64.5	32.4	29.2	23.1	25.8	-	0.0	-
Yamamoto et al., 2009	-	0.0	-	17.1	86.5	28.5	22.7	73.4	34.7	30.3	48.4	37.3
Yasuhara et al., 1998	-	0.0	-	-	0.0	-	-	0.0	-	20.1	69.8	31.2
**Class in the Steady-State**												
Lim et al., 2014	-	0.0	-	-	0.0	-	-	0.0	-	-	0.0	-
Llopis-Salvia et al., 2006	-	0.0	-	-	0.0	-	-	0.0	-	-	0.0	-
Moore et al., 2016	40.2	19.4	26.2	42.5	36.9	39.5	43.9	39.3	41.5	49.6	33.6	40.0
Mulla et al., 2005	-	0.0	-	-	0.0	-	-	0.0	-	24.0	41.0	30.3
Okada et al., 2018	14.3	93.4	24.8	16.3	83.2	27.3	20.0	72.3	31.3	24.8	84.7	38.3
Purwonugroho et al., 2012	-	0.0	-	-	0.0	-	23.2	21.0	22.0	27.9	16.9	21.1
Sánchez et al., 2010	-	0.0	-	21.3	31.0	25.2	23.1	27.9	25.3	-	0.0	-
Yamamoto et al., 2009	19.3	38.3	25.7	22.3	35.4	27.4	19.3	45.7	27.1	23.0	34.4	27.5
Yasuhara et al., 1998	-	0.0	-	-	0.0	-	-	0.0	-	29.7	32.4	31.0

Table S6. The confusion matrix of the random forest (RF) model in each scenario.

	Actual Class (%)	Lim et al., 2014	Llopis-Sal-via et al., 2006	Moore et al., 2016	Mulla et al., 2005	Okada et al., 2018	Pur-wonugroho et al., 2012	Sanchez et al., 2010	Yamamoto et al., 2009	Yasuhara et al., 1998
**Trough Sampling at Single Dose**										
Predicted Class	**Lim et al., 2014**	1.3	1.0	0.5	0.7	1.2	0.7	1.0	1.0	0.8
	**Llopis-Salvia et al., 2006**	2.7	4.4	1.4	1.3	1.9	1.5	1.4	2.8	1.6
	**Moore et al., 2016**	0.3	0.2	4.5	1.2	0.1	1.2	0.5	0.1	0.4
	**Mulla et al., 2005**	0.4	0.4	0.6	1.5	0.3	0.7	1.2	0.5	0.9
	**Okada et al., 2018**	2.7	2.4	1.1	1.9	4.0	1.5	2.4	2.8	2.9
	**Purwonugroho et al., 2012**	0.1	0.1	0.4	0.5	0.1	2.2	0.0	0.1	0.1
	**Sánchez et al., 2010**	1.7	0.9	1.3	2.4	1.4	1.5	2.5	1.4	1.8
	**Yamamoto et al., 2009**	0.9	0.9	0.4	0.5	1.1	0.6	0.6	1.2	0.8
	**Yasuhara et al., 1998**	1.1	0.8	0.9	1.1	1.0	1.1	1.4	1.2	1.8
**Trough Sampling at Steady-State**										
Predicted Class	**Lim et al., 2014**	1.0	0.6	0.7	0.6	0.7	0.8	0.9	0.7	0.6
	**Llopis-Salvia et al., 2006**	1.8	2.3	1.3	1.2	1.3	1.1	1.4	1.9	1.5
	**Moore et al., 2016**	0.7	0.6	3.4	0.7	0.1	1.1	0.3	0.6	0.5
	**Mulla et al., 2005**	1.6	0.9	1.1	3.0	1.9	1.9	1.6	1.1	1.8
	**Okada et al., 2018**	1.7	1.8	1.1	2.0	2.8	1.9	2.0	1.6	2.0
	**Purwonugroho et al., 2012**	0.6	0.5	0.4	0.4	0.6	0.7	0.8	0.6	0.6
	**Sánchez et al., 2010**	1.8	1.6	1.0	1.7	1.6	1.6	2.3	1.2	1.1
	**Yamamoto et al., 2009**	1.4	1.9	1.2	0.8	1.1	1.1	1.2	2.2	1.8
	**Yasuhara et al., 1998**	0.6	0.9	0.9	0.8	1.0	0.7	0.6	1.3	1.3
**Peak and Trough Sampling at Single Dose**										
Predicted Class	**Lim et al., 2014**	3.1	0.9	0.1	0.2	1.0	1.0	0.3	0.7	0.9
	**Llopis-Salvia et al., 2006**	1.5	3.1	1.1	1.2	1.1	1.1	1.1	1.4	1.1
	**Moore et al., 2016**	0.4	0.4	5.4	1.3	0.2	1.1	0.9	0.4	0.8
	**Mulla et al., 2005**	0.5	0.8	0.9	2.1	0.4	0.7	1.5	0.6	0.8
	**Okada et al., 2018**	2.2	2.4	1.0	1.3	4.6	1.4	1.4	3.2	2.0
	**Purwonugroho et al., 2012**	0.2	0.3	0.2	0.6	0.2	3.0	0.2	0.3	0.4
	**Sánchez et al., 2010**	0.9	1.2	1.3	3.0	0.7	0.8	4.2	0.4	2.0
	**Yamamoto et al., 2009**	2.0	1.9	0.8	0.9	2.7	1.6	0.8	3.9	1.8
	**Yasuhara et al., 1998**	0.4	0.1	0.3	0.5	0.4	0.4	0.7	0.4	1.4
**Peak and Trough Sampling at Steady-State**										
P	**Lim et al., 2014**	1.8	0.9	0.1	0.2	0.5	0.8	0.5	0.5	0.6

	**Llopis-Salvia et al., 2006**	0.8	1.1	0.4	0.3	0.5	0.5	0.6	0.6	0.5
	**Moore et al., 2016**	1.1	0.9	5.2	0.8	0.3	1.1	0.8	0.9	0.9
	**Mulla et al., 2005**	1.2	1.3	1.8	3.7	1.4	2.1	2.0	0.7	1.6
	**Okada et al., 2018**	2.2	1.9	0.8	2.4	4.8	2.5	1.8	2.6	2.2
	**Purwonugroho et al., 2012**	0.5	0.5	0.3	0.4	0.5	1.4	0.5	0.6	0.3
	**Sánchez et al., 2010**	1.2	1.6	0.8	1.9	0.7	0.9	3.4	0.4	1.5
	**Yamamoto et al., 2009**	1.9	2.5	1.2	1.2	2.0	1.7	1.2	4.3	2.1
	**Yasuhara et al., 1998**	0.5	0.3	0.4	0.2	0.4	0.2	0.2	0.4	1.3
**Peak, Mid, and Trough Sampling at Single Dose**										
**Predicted Class**	**Lim et al., 2014**	3.0	0.8	0.1	0.1	0.3	0.7	0.2	0.5	0.9
	**Llopis-Salvia et al., 2006**	1.2	3.8	0.2	0.6	0.3	0.9	0.9	1.1	1.5
	**Moore et al., 2016**	0.4	0.4	7.9	2.0	0.7	0.6	0.8	0.5	0.5
	**Mulla et al., 2005**	0.5	0.3	0.9	3.2	0.6	0.8	1.6	0.2	0.4
	**Okada et al., 2018**	2.0	1.3	1.4	1.8	6.9	1.2	1.4	1.2	0.9
	**Purwonugroho et al., 2012**	0.3	0.2	0.0	0.5	0.1	4.6	0.1	0.1	0.3
	**Sánchez et al., 2010**	0.7	0.8	0.1	1.7	0.4	0.6	4.0	0.3	1.6
	**Yamamoto et al., 2009**	2.2	2.6	0.4	0.7	1.7	1.0	1.0	6.5	2.6
	**Yasuhara et al., 1998**	0.7	0.9	0.0	0.5	0.1	0.7	1.0	0.6	2.5
**Peak, Mid, and Trough Sampling at Steady-State**										
**Predicted Class**	**Lim et al., 2014**	1.9	0.8	0.0	0.1	0.4	0.5	0.5	0.5	0.7
	**Llopis-Salvia et al., 2006**	1.1	1.8	0.1	0.3	0.3	0.7	0.9	0.7	1.0
	**Moore et al., 2016**	1.3	1.2	7.4	1.3	1.1	1.1	1.1	1.1	0.7
	**Mulla et al., 2005**	0.9	0.9	1.5	3.9	1.2	1.3	1.7	0.6	0.9
	**Okada et al., 2018**	1.8	1.2	0.9	2.2	4.9	1.3	1.6	1.6	1.4
	**Purwonugroho et al., 2012**	0.6	0.7	0.2	0.5	0.5	3.0	0.4	0.7	0.6
	**Sánchez et al., 2010**	1.0	1.3	0.1	1.1	0.4	0.6	3.0	0.3	1.3
	**Yamamoto et al., 2009**	1.8	2.5	0.7	1.2	2.1	2.0	1.3	5.1	2.3
	**Yasuhara et al., 1998**	0.7	0.7	0.1	0.4	0.3	0.6	0.5	0.5	2.2
**One-hour Interval Sampling at Single Dose**										
**Predicted Class**	**Lim et al., 2014**	4.4	0.9	0.0	0.2	0.2	0.3	0.1	0.2	0.8
	**Llopis-Salvia et al., 2006**	1.9	5.8	0.0	0.4	0.1	0.3	0.7	0.4	1.0
	**Moore et al., 2016**	0.1	0.0	10.5	0.5	0.1	0.0	0.1	0.3	0.1
	**Mulla et al., 2005**	0.4	0.3	0.0	7.0	0.5	0.6	1.4	0.2	0.2
	**Okada et al., 2018**	1.7	0.8	0.3	1.0	9.8	0.1	0.3	0.7	0.2
	**Purwonugroho et al., 2012**	0.0	0.0	0.0	0.3	0.0	9.1	0.0	0.0	0.0
	**Sánchez et al., 2010**	0.7	0.7	0.0	1.1	0.0	0.4	6.9	0.2	1.6

	**Yamamoto et al., 2009**	1.2	1.1	0.2	0.3	0.3	0.0	0.2	8.9	1.1
	**Yasuhara et al., 1998**	0.7	1.4	0.0	0.2	0.0	0.2	1.3	0.3	6.1
**One-hour Interval Sampling at Steady-State**										
**Predicted Class**	**Lim et al., 2014**	3.2	1.0	0.0	0.2	0.2	0.2	0.3	0.1	0.6
	**Llopis-Salvia et al., 2006**	1.4	2.9	0.0	0.1	0.0	0.4	1.0	0.2	1.2
	**Moore et al., 2016**	0.1	0.1	10.0	0.8	0.2	0.2	0.2	0.7	0.1
	**Mulla et al., 2005**	1.0	0.5	0.2	6.2	0.9	1.2	1.3	0.4	0.5
	**Okada et al., 2018**	2.2	1.2	0.3	1.5	7.9	0.9	0.7	1.2	0.6
	**Purwonugroho et al., 2012**	0.2	0.4	0.1	0.4	0.2	6.0	0.2	0.4	0.2
	**Sánchez et al., 2010**	1.2	2.2	0.0	0.7	0.1	0.5	5.9	0.1	1.9
	**Yamamoto et al., 2009**	1.2	1.6	0.5	1.1	1.7	1.3	0.5	7.8	1.4
	**Yasuhara et al., 1998**	0.6	1.2	0.0	0.1	0.0	0.4	1.1	0.2	4.7

Table S7. The precision, recall, and F1-Score of the random forest (RF) model in each scenario.

Sampling Scenarios	Trough (%)			Peak and Trough (%)			Peak, Mid, and Trough (%)			One-hour Interval (%)		
**Measures**	**Preci-sion**	**Recall**	**F1-Score**	**Preci-sion**	**Recall**	**F1-Score**	**Preci-sion**	**Recall**	**F1-Score**	**Preci-sion**	**Recall**	**F1-Score**
**Class in the Single Dose**												
Lim et al., 2014	15.6	11.6	13.3	37.7	27.6	31.9	45.2	27.4	34.1	61.9	39.4	48.1
Llopis-Salvia et al., 2006	23.0	39.3	29.0	24.4	27.6	25.9	36.4	34.4	35.4	54.6	52.5	53.5
Moore et al., 2016	53.1	40.6	46.0	49.7	48.7	49.2	57.4	71.2	63.5	89.3	94.6	91.9
Mulla et al., 2005	23.0	13.7	17.2	25.4	18.7	21.6	37.5	29.2	32.8	64.8	63.1	63.9
Okada et al., 2018	18.5	36.4	24.6	23.5	41.0	29.8	38.1	62.4	47.3	65.7	88.0	75.2
Purwonugroho et al., 2012	61.5	20.0	30.2	56.7	27.2	36.8	74.5	41.8	53.6	96.6	82.0	88.7
Sánchez et al., 2010	16.9	22.7	19.4	29.3	38.0	33.1	39.0	35.9	37.4	59.1	62.5	60.8
Yamamoto et al., 2009	17.0	10.7	13.1	23.6	34.7	28.1	34.7	58.3	43.5	66.6	80.5	72.9
Yasuhara et al., 1998	17.2	15.9	16.5	30.0	12.8	18.0	35.6	22.4	27.5	59.7	54.7	57.1
**Class in the Steady-State**												
Lim et al., 2014	14.6	8.6	10.8	29.5	16.1	20.8	35.6	17.0	23.0	54.7	28.4	37.4
Llopis-Salvia et al., 2006	16.7	20.8	18.5	20.6	9.9	13.4	26.2	16.1	19.9	40.1	25.8	31.4
Moore et al., 2016	42.7	31.0	35.9	43.5	47.0	45.2	45.6	66.4	54.0	80.5	89.7	84.9
Mulla et al., 2005	20.4	27.3	23.4	23.3	33.2	27.4	30.2	35.4	32.6	51.0	55.8	53.3
Okada et al., 2018	16.5	25.0	19.9	22.6	43.4	29.7	29.0	44.5	35.1	47.3	70.8	56.7
Purwonugroho et al., 2012	14.1	6.5	8.9	28.2	12.7	17.5	41.3	27.4	33.0	74.5	54.1	62.7
Sánchez et al., 2010	16.7	21.0	18.6	27.3	30.7	28.9	33.2	27.4	30.0	47.2	53.0	49.9
Yamamoto et al., 2009	17.0	19.5	18.2	23.7	38.5	29.3	26.7	45.7	33.7	45.8	70.1	55.4
Yasuhara et al., 1998	16.3	11.9	13.8	33.2	11.5	17.1	36.6	19.9	25.8	55.9	42.1	48.0

Table S8. The confusion matrix of the XGBoost model in each scenario.

	Actual Class (%)	Lim et al., 2014	Llopis-Salvia et al., 2006	Moore et al., 2016	Mulla et al., 2005	Okada et al., 2018	Purwonugroho et al., 2012	Sanchez et al., 2010	Yamamoto et al., 2009	Yasuhara et al., 1998
**Trough Sampling at Single Dose**										
Predicted Class	**Lim et al., 2014**	0.6	0.4	0.2	0.3	0.4	0.3	0.4	0.4	0.3
	**Llopis-Salvia et al., 2006**	2.9	4.6	1.5	1.3	1.9	1.5	1.3	3.1	1.7
	**Moore et al., 2016**	0.3	0.2	4.5	1.0	0.0	1.2	0.5	0.1	0.4
	**Mulla et al., 2005**	0.1	0.2	0.4	1.2	0.1	0.6	0.7	0.2	0.5
	**Okada et al., 2018**	4.5	3.9	1.8	2.9	6.3	2.5	4.0	4.2	4.4
	**Purwonugroho et al., 2012**	0.0	0.1	0.3	0.4	0.0	2.2	0.0	0.1	0.0
	**Sánchez et al., 2010**	1.9	1.2	1.5	2.8	1.5	1.7	3.3	1.6	1.9
	**Yamamoto et al., 2009**	0.3	0.3	0.2	0.1	0.3	0.4	0.1	0.5	0.2
	**Yasuhara et al., 1998**	0.4	0.3	0.7	1.1	0.5	0.7	0.9	0.9	1.6
**Trough Sampling at Steady-State**										
Predicted Class	**Lim et al., 2014**	0.3	0.1	0.2	0.1	0.1	0.2	0.2	0.0	0.1
	**Llopis-Salvia et al., 2006**	2.0	2.7	1.5	0.9	1.1	1.6	1.8	2.3	1.5
	**Moore et al., 2016**	0.8	0.5	3.5	0.8	0.1	1.7	0.2	0.5	0.4
	**Mulla et al., 2005**	1.5	0.8	1.1	3.0	1.5	1.5	1.2	1.1	1.7
	**Okada et al., 2018**	3.1	3.3	1.8	3.8	5.6	3.3	4.2	3.0	3.7
	**Purwonugroho et al., 2012**	0.0	0.0	0.0	0.0	0.0	0.0	0.0	0.0	0.0
	**Sánchez et al., 2010**	1.9	1.9	1.2	1.7	1.7	1.9	2.5	1.2	1.0
	**Yamamoto et al., 2009**	1.2	1.5	1.1	0.6	0.9	0.7	0.8	2.4	1.9
	**Yasuhara et al., 1998**	0.4	0.3	0.6	0.3	0.2	0.3	0.1	0.5	0.8
**Peak and Trough Sampling at Single Dose**										
Predicted Class	**Lim et al., 2014**	3.0	0.9	0.1	0.1	0.9	0.9	0.2	0.6	0.9
	**Llopis-Salvia et al., 2006**	1.6	3.4	1.1	1.2	1.0	1.0	1.1	1.4	1.0
	**Moore et al., 2016**	0.2	0.3	5.0	1.1	0.1	1.2	0.6	0.3	0.5
	**Mulla et al., 2005**	0.5	0.5	1.0	2.1	0.4	0.9	1.2	0.6	0.7
	**Okada et al., 2018**	2.6	2.6	1.5	1.8	5.7	1.8	1.9	3.8	2.3
	**Purwonugroho et al., 2012**	0.3	0.2	0.2	0.5	0.1	2.8	0.1	0.2	0.3
	**Sánchez et al., 2010**	0.9	1.3	1.3	3.0	0.7	0.9	4.9	0.3	2.3
	**Yamamoto et al., 2009**	1.9	1.8	0.6	0.9	2.1	1.4	0.7	3.8	1.9
	**Yasuhara et al., 1998**	0.3	0.1	0.2	0.3	0.1	0.2	0.4	0.2	1.1
**Peak and Trough Sampling at Steady-State**										
P	**Lim et al., 2014**	1.8	0.9	0.1	0.2	0.5	0.8	0.6	0.4	0.6

	Llopis-Salvia et al., 2006	0.6	1.0	0.4	0.2	0.2	0.4	0.3	0.4	0.5
	Moore et al., 2016	1.1	0.8	4.9	0.7	0.3	1.2	0.7	0.7	0.9
	Mulla et al., 2005	1.4	1.5	2.0	4.1	1.6	1.6	2.2	1.0	1.8
	Okada et al., 2018	2.4	2.3	1.0	2.7	5.3	2.8	2.0	3.0	2.5
	Purwonugroho et al., 2012	0.4	0.3	0.3	0.4	0.3	1.6	0.4	0.5	0.3
	Sánchez et al., 2010	1.2	1.6	0.8	1.7	0.7	0.9	3.5	0.4	1.4
	Yamamoto et al., 2009	1.9	2.4	1.2	1.0	1.9	1.7	1.3	4.4	2.0
	Yasuhara et al., 1998	0.4	0.3	0.4	0.2	0.3	0.2	0.2	0.2	1.2
**Peak, Mid, and Trough Sampling at Single Dose**										
Predicted Class	Lim et al., 2014	2.9	0.7	0.0	0.1	0.3	0.7	0.2	0.4	0.9
	Llopis-Salvia et al., 2006	1.3	3.8	0.2	0.5	0.3	0.8	0.9	1.2	1.4
	Moore et al., 2016	0.4	0.5	7.9	1.9	0.8	0.6	0.9	0.7	0.4
	Mulla et al., 2005	0.5	0.3	0.9	3.2	0.6	0.9	1.5	0.2	0.4
	Okada et al., 2018	2.0	1.3	1.5	1.9	7.0	1.2	1.3	1.1	0.9
	Purwonugroho et al., 2012	0.3	0.2	0.0	0.5	0.0	4.5	0.1	0.1	0.4
	Sánchez et al., 2010	0.6	0.8	0.1	1.8	0.3	0.6	4.2	0.3	1.5
	Yamamoto et al., 2009	2.4	2.7	0.4	0.8	1.6	1.1	1.1	6.7	2.8
	Yasuhara et al., 1998	0.7	0.8	0.1	0.5	0.0	0.8	0.9	0.5	2.4
**Peak, Mid, and Trough Sampling at Steady-State**										
Predicted Class	Lim et al., 2014	1.7	0.7	0.0	0.1	0.3	0.4	0.5	0.3	0.5
	Llopis-Salvia et al., 2006	1.0	1.7	0.0	0.2	0.2	0.6	0.9	0.6	1.0
	Moore et al., 2016	1.3	1.2	7.4	1.4	1.1	1.1	1.1	1.0	0.8
	Mulla et al., 2005	1.0	0.8	1.6	4.0	1.2	1.3	1.8	0.7	1.0
	Okada et al., 2018	1.7	1.3	0.9	2.1	5.1	1.4	1.7	1.5	1.5
	Purwonugroho et al., 2012	0.6	0.5	0.2	0.5	0.3	3.1	0.4	0.6	0.5
	Sánchez et al., 2010	1.1	1.3	0.1	1.0	0.3	0.6	2.9	0.2	1.2
	Yamamoto et al., 2009	2.1	2.9	0.7	1.4	2.4	2.1	1.4	5.7	2.5
	Yasuhara et al., 1998	0.6	0.6	0.1	0.3	0.2	0.4	0.4	0.5	2.1
**One-hour Interval Sampling at Single Dose**										
Predicted Class	Lim et al., 2014	4.7	0.9	0.0	0.3	0.1	0.2	0.1	0.1	0.6
	Llopis-Salvia et al., 2006	1.7	5.6	0.0	0.3	0.1	0.1	0.6	0.1	0.9
	Moore et al., 2016	0.0	0.0	11.0	0.5	0.2	0.0	0.1	0.3	0.0
	Mulla et al., 2005	0.3	0.3	0.0	7.5	0.3	0.9	1.4	0.1	0.3
	Okada et al., 2018	1.6	0.5	0.0	0.8	9.8	0.0	0.2	0.3	0.1
	Purwonugroho et al., 2012	0.0	0.0	0.0	0.0	0.0	9.2	0.0	0.0	0.0
	Sánchez et al., 2010	0.5	0.7	0.0	1.0	0.0	0.3	7.0	0.1	1.2

	**Yamamoto et al., 2009**	1.2	1.5	0.1	0.4	0.6	0.0	0.3	10.0	1.1
	**Yasuhara et al., 1998**	1.0	1.6	0.0	0.2	0.0	0.3	1.4	0.1	6.8
**One-hour Interval Sampling at Steady-State**										
**Predicted Class**	**Lim et al., 2014**	3.4	1.0	0.0	0.1	0.2	0.3	0.4	0.0	0.6
	**Llopis-Salvia et al., 2006**	1.2	3.0	0.0	0.1	0.0	0.4	1.3	0.0	1.2
	**Moore et al., 2016**	0.1	0.1	10.7	1.0	0.3	0.3	0.1	0.7	0.1
	**Mulla et al., 2005**	0.7	0.6	0.0	6.4	0.9	1.1	1.4	0.4	0.5
	**Okada et al., 2018**	2.2	1.2	0.0	1.3	7.2	0.5	0.5	0.8	0.5
	**Purwonugroho et al., 2012**	0.4	0.7	0.1	0.3	0.3	6.9	0.3	0.7	0.4
	**Sánchez et al., 2010**	1.0	1.6	0.0	0.5	0.1	0.3	5.4	0.0	1.3
	**Yamamoto et al., 2009**	1.3	1.4	0.2	1.2	2.1	1.0	0.5	8.5	1.3
	**Yasuhara et al., 1998**	0.8	1.4	0.0	0.1	0.0	0.4	1.1	0.1	5.2

Table S9. The precision, recall, and F1-Score of XGBoost in each scenario.

Sampling Scenarios	Trough (%)			Peak and Trough (%)			Peak, Mid, and Trough (%)			One-hour Interval (%)		
**Measures**	**Precision**	**Recall**	**F1-Score**	**Precision**	**Recall**	**F1-Score**	**Precision**	**Recall**	**F1-Score**	**Precision**	**Recall**	**F1-Score**
**Class in the Single Dose**												
Lim et al., 2014	17.2	5.0	7.7	38.7	26.8	31.7	46.5	26.1	33.4	65.8	42.1	51.3
Llopis-Salvia et al., 2006	23.3	41.2	29.7	26.5	30.3	28.3	36.6	34.6	35.6	59.4	50.3	54.5
Moore et al., 2016	54.0	40.4	46.2	54.4	45.4	49.5	56.3	71.1	62.8	89.5	98.6	93.8
Mulla et al., 2005	29.1	10.4	15.3	26.8	19.1	22.3	37.7	28.6	32.5	67.4	67.6	67.5
Okada et al., 2018	18.1	56.5	27.4	23.8	51.6	32.6	38.7	63.2	48.0	72.9	88.6	80.0
Purwonugroho et al., 2012	67.8	19.6	30.4	58.5	24.9	34.9	74.7	40.8	52.8	99.0	82.9	90.3
Sánchez et al., 2010	18.7	29.4	22.9	31.1	44.0	36.5	41.0	38.1	39.5	64.7	63.2	63.9
Yamamoto et al., 2009	20.1	4.4	7.2	25.5	34.4	29.3	34.0	60.0	43.4	65.7	89.9	75.9
Yasuhara et al., 1998	23.3	14.8	18.1	37.3	9.8	15.5	36.1	21.6	27.0	60.3	60.8	60.5
**Class in the Steady-State**												
Lim et al., 2014	24.0	2.5	4.5	31.2	16.2	21.3	37.4	15.7	22.1	56.4	30.5	39.6
Llopis-Salvia et al., 2006	17.4	24.2	20.2	24.9	9.0	13.2	27.8	15.6	20.0	40.9	26.9	32.4
Moore et al., 2016	41.9	31.9	36.2	43.6	44.0	43.8	45.2	66.7	53.9	80.1	96.3	87.5
Mulla et al., 2005	22.2	26.9	24.3	23.9	36.8	29.0	29.4	35.6	32.2	53.0	57.5	55.2
Okada et al., 2018	17.7	50.2	26.1	22.2	48.0	30.3	29.7	45.9	36.1	50.6	65.2	57.0
Purwonugroho et al., 2012	5.9	0.1	0.2	35.7	14.7	20.8	45.8	27.6	34.5	68.0	61.7	64.7
Sánchez et al., 2010	16.6	22.4	19.1	28.8	31.6	30.1	33.1	26.3	29.3	52.5	48.3	50.3
Yamamoto et al., 2009	21.4	21.4	21.4	24.8	39.6	30.5	26.6	50.9	35.0	48.3	76.2	59.1
Yasuhara et al., 1998	22.5	7.3	11.0	36.1	10.7	16.5	40.0	19.1	25.8	56.6	46.5	51.1

Table S10. The mean percent error (MPE) and relative root mean squared error ($rRMSE$) of the predicted AUC relative to the true AUC of each simulation scenario using objective function values (OFVs) for model selection and weighted averaging.

Measures		MPE (%)				$rRMSE$ (%)			
**Test Model**	**Classification**	**Trough**	**Peak and Trough**	**Peak, Mid, and Trough**	**One-hour Interval**	**Trough**	**Peak and Trough**	**Peak, Mid, and Trough**	**One-hour Interval**
**Internal Validation**									
**Single Dose**	Selection	-2.65	-1.35	1.16	1.70	19.02	16.50	13.90	8.31
	Weighted Average	0.37	-0.75	1.52	1.72	18.70	16.19	13.69	8.25
**Steady State**	Selection	-4.41	-3.04	-1.38	-1.41	16.79	13.11	10.46	5.65
	Weighted Average	-1.81	-2.63	-1.10	-1.40	16.10	12.90	10.20	5.60
**External Validation**									
**Single Dose**	Selection	8.16	1.07	3.86	1.43	31.68	22.72	19.86	11.38
	Weighted Average	10.91	1.97	4.16	1.42	32.03	22.68	19.61	11.28
**Steady State**	Selection	2.27	-1.11	0.39	-0.61	23.54	17.53	14.78	7.74
	Weighted Average	4.41	-0.86	0.46	-0.64	23.92	17.38	14.54	7.68

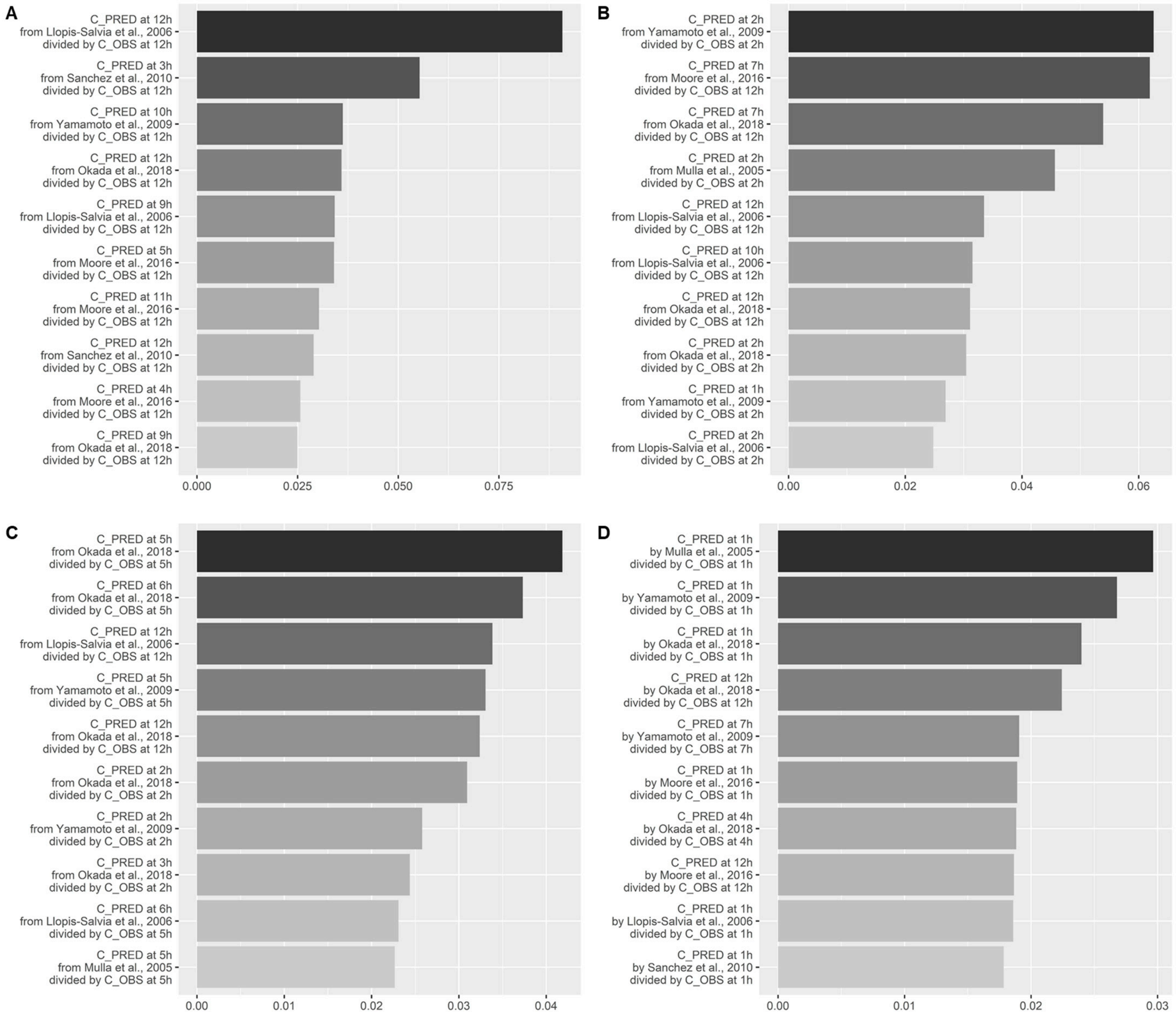

Figure S1. The feature importance plot of the XGBoost model in a single dose. The x-axis represents the XGBoost importance value of the feature, whereas the y-axis represents the concentration used for feature creation. Out of the 108 features created, 10 features with the highest importance values are presented. (**A**) Trough, (**B**) peak and trough, (**C**) peak, mid, and trough, and (**D**) one-hour interval sampling.

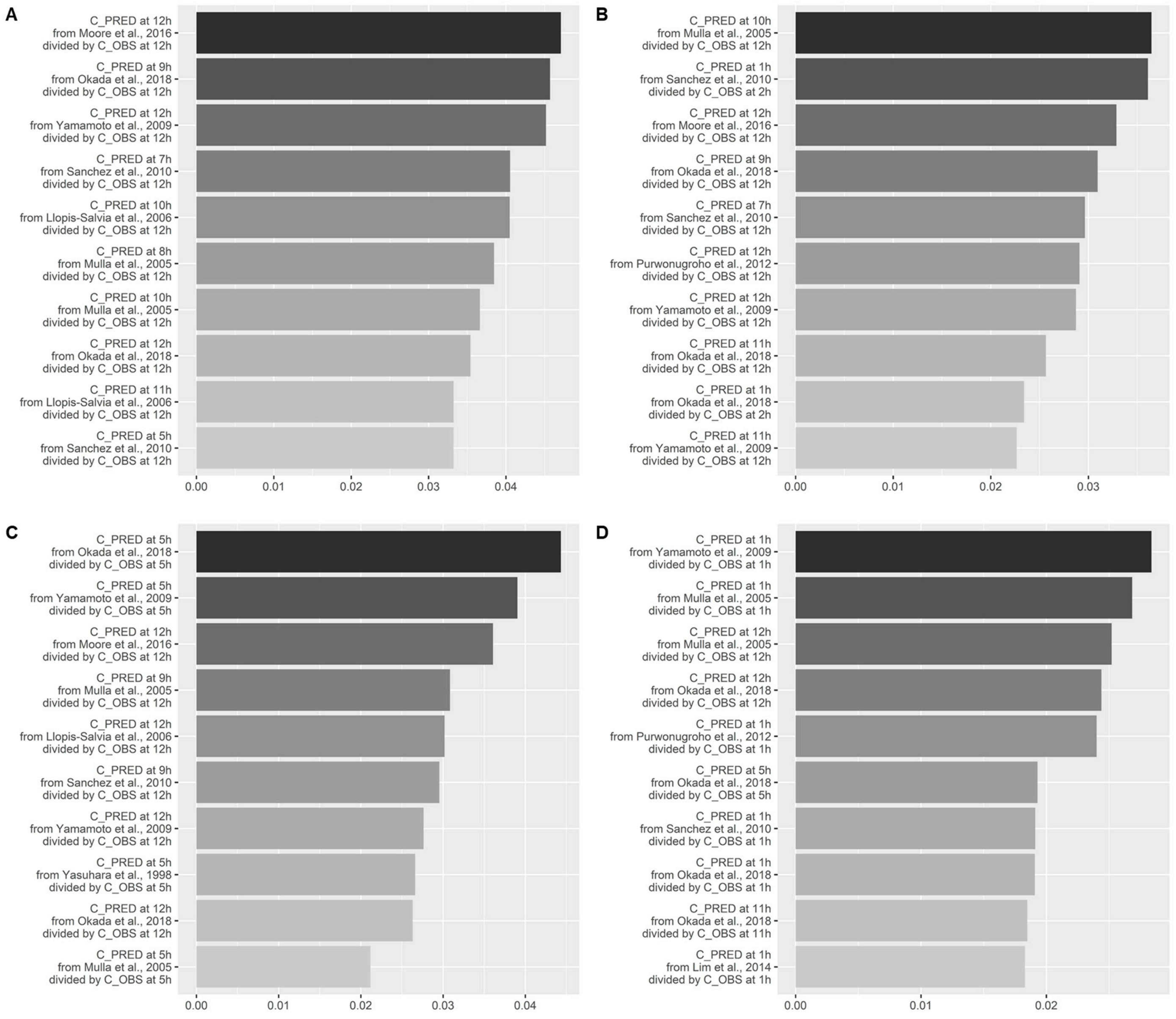

Figure S2. The feature importance plot of the XGBoost model in the steady state. The x-axis represents the XGBoost importance value of the feature, whereas the y-axis represents the concentration used for feature creation. Out of the 108 features created, 10 features with the highest importance values are presented. (**A**) Trough, (**B**) peak and trough, (**C**) peak, mid, and trough, and (**D**) one-hour interval sampling.
